# Bronchial smooth muscle extracellular vesicles interfere with bronchial epithelium metabolism and function in asthma

**DOI:** 10.1016/j.isci.2025.112546

**Published:** 2025-04-29

**Authors:** Elisa Celle, Amine Chahin, Fabien Beaufils, Guillaume Cardouat, Edmée Eyraud, Clément Bouchet, Marilyne Campagnac, Olga Ousova, Hugues Begueret, Matthieu Thumerel, Rémi Dubois, Jean-William Dupuy, Thierry Leste-Lasserre, Sabrina Lacomme, Nina Lager-Lachaud, Floriant Bellvert, Roger Marthan, Pierre-Olivier Girodet, Patrick Berger, Thomas Trian, Pauline Esteves

**Affiliations:** 1 Univ-Bordeaux, Centre de Recherche Cardio-thoracique de Bordeaux, U1045, Département de Pharmacologie, Bordeaux Proteome, TBM-Core, US5, UAR 3427, OncoProt, CIC 1401, 33000 Bordeaux, France; 2 INSERM, Centre de Recherche Cardio-thoracique de Bordeaux U1045, Plateforme Transcriptome Neurocentre Magendie U1215, TBM-Core, US5, UAR 3427, OncoProt, BIC, UAR 3420, 33000 Bordeaux, France; 3 CHU de Bordeaux, Service d’exploration fonctionnelle respiratoire, Service de pharmacologie, CIC 1401, Service de chirurgie thoracique, 33604 Pessac, France; 4 CNRS, TBM-Core, US5, UAR 3427, OncoProt, BIC, UAR 3420, 33000 Bordeaux, France; 5 Université de Toulouse, CNRS 5504, INRA 792, INSA Toulouse, Toulouse Biotechnology Institute, Bio & Chemical Engineering, MetaToul, Toulouse, France

**Keywords:** Pathology, Cell biology

## Abstract

Bronchial smooth muscle (BSM) remodeling is an important feature of severe asthma pathophysiology. We previously showed that asthmatic BSM is metabolically different and increased rhinovirus (RV) replication rate, the main trigger of severe asthma exacerbations. Extracellular vesicles (EVs) are the key mediator in cell-cell communication, but the role of BSM cells-derived EVs on bronchial epithelial has never been investigated in asthma. Using severe asthmatic and non-asthmatic tissue collection, we show that asthmatic BSM cells are able to produce a greater amount of EVs containing metabolites involved in bioenergetics. We study the bronchial epithelium energetic rewiring following stimulation with asthmatic BSM cells-derived EVs. Modifications of bronchial epithelium metabolic behavior were associated with an increased ATP production and a breakdown of bronchial epithelium barrier function such as ciliary beating frequency and efficiency. Finally, we show that asthmatic BSM cells-derived EVs increased RV replication in bronchial epithelium following RV infection.

## Highlights

• Asthmatic BSM cells produce an increased number of extracellular vesicles (EVs)
• Asthmatic BSM cells-derived EVs rewire cellular energetic metabolism of bronchial epithelium
• Metabolic modifications are associated to a decreased barrier function
• Asthmatic BSM cells-derived EVs increase rhinovirus infection

## Graphical abstract

**Figure:**
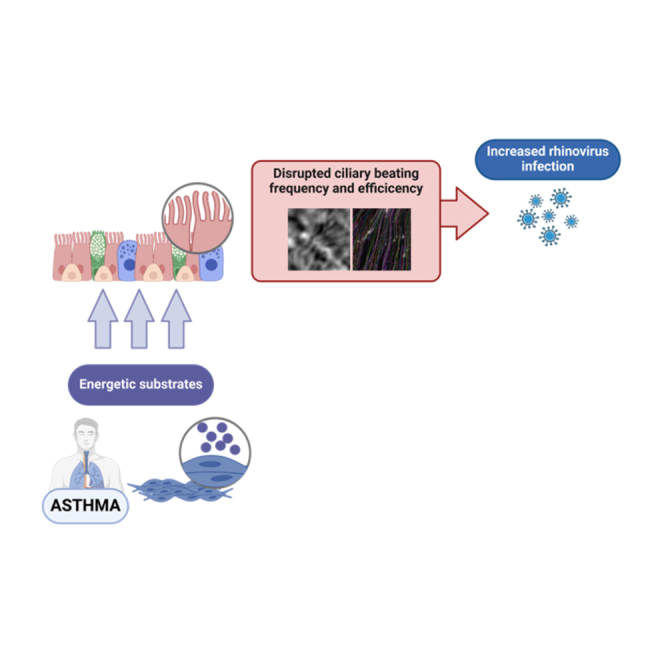

## Introduction

Asthma is the most common chronic airway disease that affects 6–20% of the population of Western European countries, causing substantial health and economic burdens worldwide.^1^ Severe asthma, defined according to the ATS/ERS task force, affects 3–5% of all asthmatic patients but is responsible for a large proportion of resource expenditures.^2^ Histologically, bronchi from severe asthmatic patients are remodeled and harbor various structural changes characterized by bronchial epithelial abnormalities, such as metaplasia, thickened epithelium and reticular basement membrane, goblet cell hyperplasia or mucus hypersecretion,^3^ increased subepithelial membrane thickness, mucous gland hypertrophy, neo-angiogenesis, and increased bronchial smooth muscle (BSM) mass.^4^

The most crucial feature of bronchial remodeling appears to be BSM remodeling, since increased BSM mass is associated with both lower lung function^5^^,^^6^ and higher exacerbation rate,^7^^,^^8^^,^^9^ which are of poor prognostic value in asthma. BSM remodeling can begin very early in life, often at preschool age, and predicts the persistence of asthma in school-age children.^8^^,^^9^ Clinically, the reduction of asthma exacerbations remains a major unmet need in patients with severe asthma, whereas recent advances in new biologic therapies have significantly decreased the exacerbation rate.^10^^,^^11^^,^^12^^,^^13^^,^^14^ However, patients on biologics continued to present some exacerbations, albeit at a lower rate.^10^^,^^12^^,^^13^^,^^14^^,^^15^

The main role of the bronchial epithelium is to protect the airways against aerocontaminants responsible for asthma exacerbation. Indeed, viral infection of the bronchial epithelium has been implicated in 80% of asthma exacerbations in children and adolescents and in 60% of those in adults.^16^ In each case, the human rhinovirus was the dominant viral pathogen, and rhinovirus caused 60% of all virus-induced asthma exacerbations.^16^ The susceptibility of the bronchial epithelium to rhinovirus infection appears to be increased in asthmatic patients^17^ but the mechanism remains unclear even though intrinsic modifiers, such as upstream activators of interferon production, have been demonstrated.^18^^,^^19^ In addition, BSM increased bronchial epithelium susceptibility to rhinovirus by increasing rhinovirus replication within the bronchial epithelium layer through a CCL20/PKR signaling pathway.^20^

Bronchial epithelium is heterogeneous and is indeed mainly composed by ciliated, basal, club, and goblet cells. Both ciliary beating and mucus production are involved in the innate defense against pathogens. Indeed, mucus covers bronchial epithelium and traps inhaled pathogens which are then moved away from the lung using ciliary beating.^21^ These bronchial epithelial functions are high energy consuming and the ATP production is a crucial limiting factor.^22^ Indeed, ATP concentration is directly associated with ciliary beating frequency^23^^,^^24^ and a recent study also highlights the indispensable need for ATP synthesis in mucus production.^25^

Several studies have shown that small extracellular vesicles (EVs), including exosomes, lead to cell-cell communication through metabolites, proteins, lipids, RNA and even mitochondrial DNA transfer.^26^^,^^27^^,^^28^ Such an important role of EVs have been described in different inflammatory pathways, such as cancer or inflammatory diseases.^29^^,^^30^ In the complex asthma pathophysiology, Mazzeo et al., described the potential role of eosinophils-derived exosomes, in the maintenance of asthma inflammation.^31^ Exosomes have also been associated with metabolism modifications. For example, exosomes from cancer-associated fibroblasts can increase glycolysis and reduce OXPHOS capacities in pancreatic cells,^32^ and those from oligodendrocytes can transfer SIRT2 to axons where it could participate to mitochondrial capacity.^33^ In asthma, there are few studies that investigate the role of EVs. However, a study from Hough et al. has characterized a unique lipid signature of EVs purified from asthmatic’ bronchoalveolar lavage and show that airways’ myeloid cells can communicate with lymphocytes and activate mitochondrial metabolism which, in terms, contribute to generate ROS signaling, participating in lymphocytes activation.^34^

The goal of the present study was thus to assess the role of asthmatic BSM cells-derived small EVs, including exosomes, in bronchial epithelium physiology and response to rhinovirus infection. Using primary human cell culture of bronchial epithelial and BSM cells, we showed that asthmatic BSM cells-derived EVs increased metabolites delivery to bronchial epithelium and rewire the cellular energetic metabolism of the host cell. Moreover, we demonstrated that such metabolic changes drastically affected bronchial epithelium function and, more precisely, ciliary beating frequency and efficiency that are responsible for rhinovirus increased infection of the bronchial epithelium.

## Results

### Patients’ characteristics

The clinical and functional characteristics of both severe asthmatic patients and non-asthmatic patients are presented in Table 1. Asthmatic patients were similar to non-asthmatic patients in terms of the sex ratio and age. Despite the heterogeneity in the non-asthmatic group, we paid special attention to enroll a population of non-asthmatic patients with normal lung function values for both FEV-1 and FVC values and without any inhaled medicines. Asthmatic patients presented similar forced expiratory volume in 1 s (FEV-1) and forced vital capacity (FVC) values compared to non-asthmatic patients, which reflected a controlled asthma.

**Table 1:** Clinical and functional characteristics of subjects

Characteristics	Non-asthma	Asthma	*p* value
No. of patients	28	15	
Sex (M/F)	15/13	6/9	NS
Age, yr	65.46 ± 2.32	57.21 ± 4.03	NS
Treatments			
ICS, no. of patients	0	15	
LABA, no. of patients	0	14	
OCS, no. of patients	0	2	
FEV1			
Liters	2.16 ± 0.10	2.25 ± 0.21	NS
FVC			
Liters	2.85 ± 0.19	3.01 ± 0.25	NS
Smoking history			
Current (no. of patients/Total of patients)	9/28	0/15	0.001
Former (no. of patients/Total of patients)	15/28	7/15	NS
Never (no. of patients/Total of patients)	4/28	8/15	0.01
Allergy		11/15	

Plus–minus values are means ± SEM. LABA, long-acting beta-agonist; ICS, inhaled corticosteroid; OCS, oral corticosteroid; FEV_1_, forced expiratory volume in 1 s; FVC, forced vital capacity. *p* values were calculated with the use of a two-sided independent t-test for variables with a parametric distribution and the Mann–Whitney U test for comparison of nonparametric variables.

### Asthmatic BSM cells produce an increased quantity of EVs

**Figure 1:**
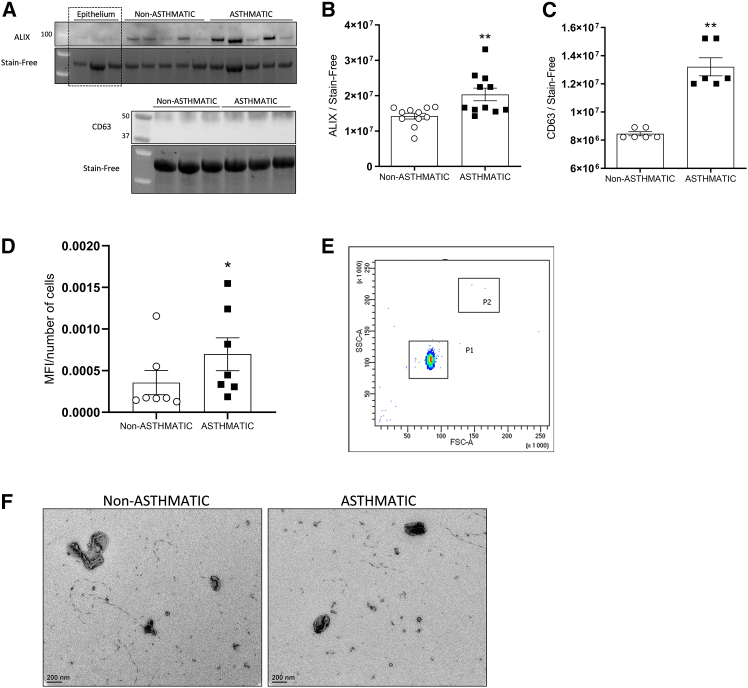
Increased production of extracellular vesicles in asthmatic BSM cells (A–C) Representative images and quantification of ALIX (*n* = 11) and CD63 (*n* = 6) protein expression in non-asthmatic (white circle) and asthmatic (black square) BSM cells-derived extracellular vesicles using western blotting. Stain-free gel technology was used for loading control expression quantification. (D) Quantification of CD63^+^ non-asthmatic (white circle, *n* = 7) and asthmatic (black square, *n* = 7) BSM cells-derived extracellular vesicles using flow cytometry. (E) Representative distribution of CD63^+^ EVs population with SSC and FCS gating. P1 represents the CD63^+^ beads population used for quantification while P2 refers to debris population. (F) Representative images of non-asthmatic and asthmatic BSM cells-derived extracellular vesicles using electronic microscopy. Data are presented as mean ± SEM. ∗*p* < 0.05 and ∗∗*p* < 0.01.

We proceed to small EVs purification, from BSM cells culture medium using standard and validated protocols, including culture of BSM cells in a cell culture media supplemented with EVs-free fetal bovine serum in order to avoid external contamination with EVs.^35^^,^^36^ Following recommended techniques for identification and quantification of exosomes,^36^ we first analyzed the expression of proteins such as CD63 (a tetraspanin protein) and Alix (a cytosolic stress protein), both described as specific for small EVs and exosomes, using western-blotting. We showed that both CD63 and Alix proteins expression from asthmatic BSM cells-derived EVs were significantly increased compared to non-asthmatic BSM cells-derived EVs (Figures 1A–1C). EVs were originated from BSM cells since we verified that both asthmatic and non-asthmatic BSM cells were able to express CD63 and Alix endogenously (Figure S1). Then, to confirm an increased amount of EVs, we proceed to their quantification using CD63 tagged magnetic beads labeled with fluorescent protein phycoerythrin (PE) suitable for flow cytometry. We showed that asthmatic BSM cells produced a significant greater amount of CD63^+^ EVs compared to non-asthmatic BSM cells (Figure 1D). Using flow cytometry experiments, we showed that the EVs populations were homogeneous in term of size distribution as indicated with SSC and FSC-dependent distribution patterns (Figure 1E). Finally, we performed transmission electronic microscopy of freshly purified EVs from BSM cells culture medium (Figure 1F). According to the literature, we observed EVs with a distribution size ranging from 50–200 nm in diameter. We did not observe any difference in terms of size distribution between EVs purified from asthmatic and those from non-asthmatic BSM cells (data not shown).

### Asthmatic BSM cells-derived EVs contained significant amounts of metabolites involved in bioenergetics

**Figure 2:**
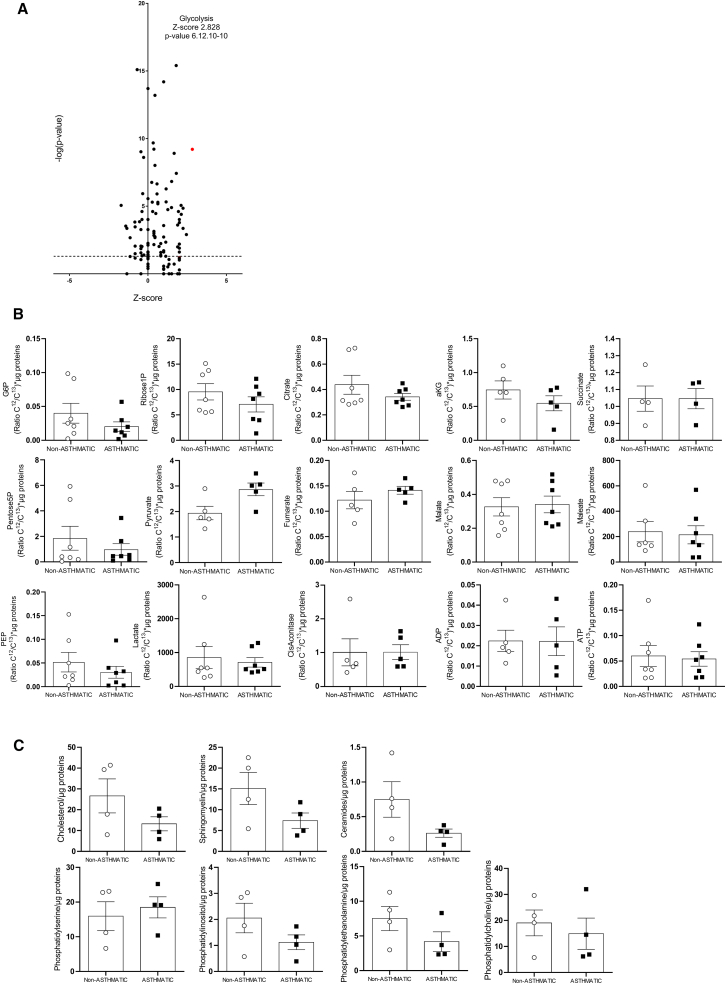
Extracellular vesicles content analysis Proteome comparison between non-asthmatic and asthmatic BSM cells-derived EVs (*n* = 3) was performed using mass spectrometry analysis associated with Ingenuity Pathway Analysis (IPA) from the raw proteomic data. The proteins with a different expression were organized based on pre-defined categories suggested by IPA. Then, the proteins with different expression were assigned to these IPA categories based on the IPA database. The categories were ranked according to their frequency of identification in the proteome [-log (*p* value)]. (A) Representative volcano plot of all the pathways identified using IPA. (B) Metabolomic analysis was performed using mass spectrometry on EVs from non-asthmatic (n = 4–7) and asthmatic (n = 5–7) BSM cells. Metabolites were represented and segmented by their belonging pathway using an heatmap. (C) Lipidomic analysis was performed using mass spectrometry on EVs from non-asthmatic (white circle, *n* = 4) and asthmatic (black square, *n* = 4) BSM cells. Concentrations were normalized to total μg of EVs proteins. Data are presented as mean ± SEM.

Since EVs have been described as, particles containing different types of substrates, we thus performed several identification analyses. Comparison of non-asthmatic and asthmatic BSM derived EVs content was performed using the same quantity of EVs. Significative increased expression of EVs by asthmatic could not be consider for comparative studies. First, we proceed to a proteomic analysis of EVs content. Raw data from the label-free proteomics approach were shared through PRIDE (under accession number PXD055968) and revealed significant modifications in the expression of 278 proteins (Figure S2). Using Ingenuity Pathway Analysis, we demonstrated significant alterations in various canonical pathways (Figure 2A; Table S1). Among all the pathways, “glycolysis” appeared as the pathway with the highest activation *Z* score of 2.828 and with an associated *p*-value 6.12.10^−10^ (Figure 2A). Among the “glycolysis” pathway, we detected 8 significative upregulated proteins expression of glycolysis pathway in asthmatic BSM cells-derived EVs (Table S2). Second, we performed a metabolic analysis of EVs content in order to identify significant increased metabolites in asthmatic BSM cells-derived EVs compared to non-asthmatic. We detected 15 metabolites either from glucose catabolism, glycolysis and the citric acid cycle (Figure 2B). Finally, using lipidomic analysis, six different lipids were identified in asthmatic and non-asthmatic BSM cells-derived EVs with similar levels of expression (Figure 2C).

### Enhanced bronchial epithelium energetic metabolism is induced by asthmatic BSM cells-derived EVs

**Figure 3:**
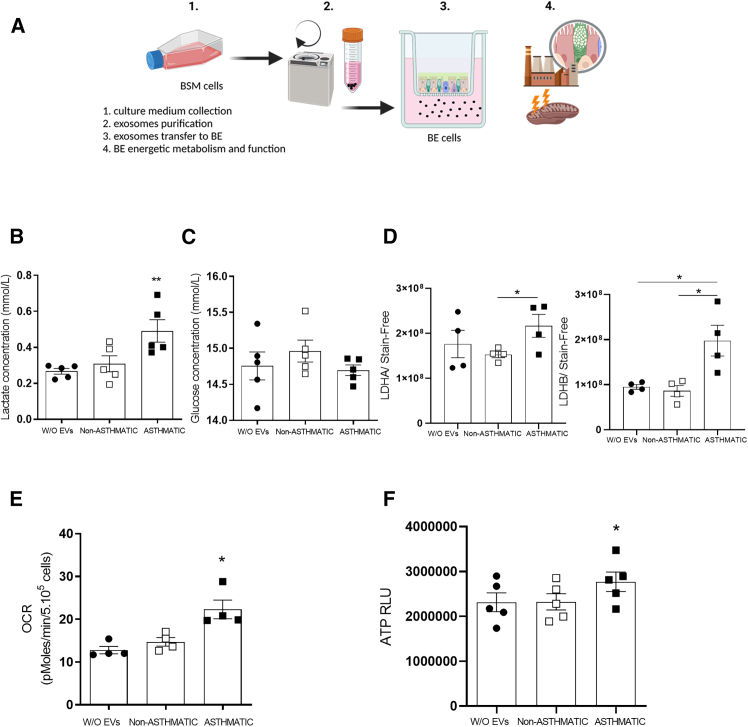
Metabolic remodeling of bronchial epithelium (A) Representative framework of BSM cells derived extracellular vesicles transfer to bronchial epithelium. Extracellular vesicles were collected and purified from either non-asthmatic and asthmatic BSM cells culture medium. Extracellular vesicles were transferred and diluted into the bronchial epithelium culture medium. Energetic metabolic changes were observed 6 h after the challenge. (B) Lactate and (C) glucose concentration was assessed in the bronchial epithelium culture medium of BE cultured without EVs (black circle, *n* = 5), non-asthmatic (white square, *n* = 5) and asthmatic (black square, *n* = 5) BSM cells-derived EVs during 6 h. (D) Quantification of LDHA and LDHB (*n* = 4) protein expression in epithelium cultured without EVs (black circle, *n* = 4), non-asthmatic (white square, *n* = 4) and asthmatic (black square, *n* = 4) BSM cells-derived EVs during 6 h using Western-blotting. (E) Oxygen consumption rate (OCR) was measured in basal condition (DMEM 5 mM glucose) bronchial epithelium cultured without EVs (black circle, *n* = 4), non-asthmatic (white square, *n* = 4) and asthmatic (black square, *n* = 4) BSM cells-derived EVs during 6 h. The respiration rate was corrected for non-mitochondrial respiration determined after the addition of antimycin and potassium cyanide. (F) Steady-state ATP content was measured in bronchial epithelium cultured without EVs (black circle, *n* = 5), non-asthmatic (white square, *n* = 5) and asthmatic (black square, *n* = 5) BSM cells-derived EVs after 6 h using a luminescence assay. Data are presented as mean ± SEM. ∗*p* < 0.05.

In order to observe the effects of a communication between BSM cells-derived EVs and the bronchial epithelium, we proceed to EVs purification and transfer them to bronchial epithelial cells (Figure 3A). All proteomic analysis were performed with non-asthmatic epithelium and were treated with EVs for 24h in order to be able to detect proteome adaptation.^37^^,^^38^^,^^39^ Raw data from the label-free proteomics approach were shared through PRIDE (under accession number PXD055949). Using proteomic analysis associated to Ingenuity Pathway Analysis and cellular function filter, we have highlighted proteins rearrangement belonging to “catabolism of macromolecules” with activation z-scores of 1.543 (*p* = 8.65.10^−63^) in bronchial epithelium challenged with asthmatic BSM cells-derived EVs compared to non-asthmatic BSM cells-derived EVs. This could suggest an enhanced consumption and degradation of molecules including polysaccharides and lipids within bronchial epithelium challenged with asthmatic EVs compared to non-asthmatic EVs. To go further, using IPA, we analyze proteome rearrangement that affect metabolism pathways. We were able to observe that proteins rearrangement occurred in “The citric acid cycle and respiratory electron transport” (48/55 molecules, *Z* score 2.59, *p* = 2.94.10^−19^), “Oxidative phosphorylation” (89/112 molecules, *Z* score 2.25, *p* = 2.24.10^−28^) and “glucose metabolism” (65/80 molecules, *Z* score 0.75, *p* = 4.42.10^−22^). We observed proteins rearrangement in pathways from mitochondrial function and glucose degradation specifically in bronchial epithelium challenged with asthmatic BSM cells-derived EVs.

To evaluate energetic metabolism, first, using nuclear magnetic resonance spectrometry on the bronchial epithelium cell culture medium, we identified the level of lactate which indicated the level of aerobic glycolysis within the bronchial epithelium 6 h after the transfer of EVs. We observed that bronchial epithelium challenged with asthmatic BSM cells-derived EVs produced a significant increased level of lactate compared to untreated bronchial epithelium and bronchial epithelium challenged with non-asthmatic BSM cells-derived EVs (Figure 3C). We attributed this increased production of lactate from bronchial epithelium to the EVs content since the level of glucose, the main energetic substrate of bronchial epithelium contained in cell culture medium was not differently absorbed between the 3 experimental conditions (Figure 3D). We measured the protein level of two isoforms of lactate dehydrogenase LDHA and LDHB. We found a significant increase in both LDHA and LDHB in epithelium challenged during 6 h with asthmatic BSM cells-derived EVs compared to that challenged with non-asthmatic BSM cells-derived EVs or in the untreated bronchial epithelium (Figures 3E; Figure S3). In parallel, functional evaluation of the mitochondrial bioenergetics revealed an increased rate of mitochondrial respiration in bronchial epithelium challenged during 6 h with asthmatic BSM cells-derived EVs compared to that challenged with non-asthmatic BSM cells-derived EVs or in the untreated bronchial epithelium (Figure 3F). The steady-state ATP content at 6 h post-transfer of EVs, was significantly increased in bronchial epithelium challenged with asthmatic BSM cells-derived EVs compared to that challenged with non-asthmatic BSM cells-derived EVs or untreated bronchial epithelium (Figure 3G). Interestingly, ATP content was not different 24 h after the challenge with either asthmatic or non-asthmatic BSM cells-derived EVs compared to untreated bronchial epithelium (Figure S4).

**Figure 4:**
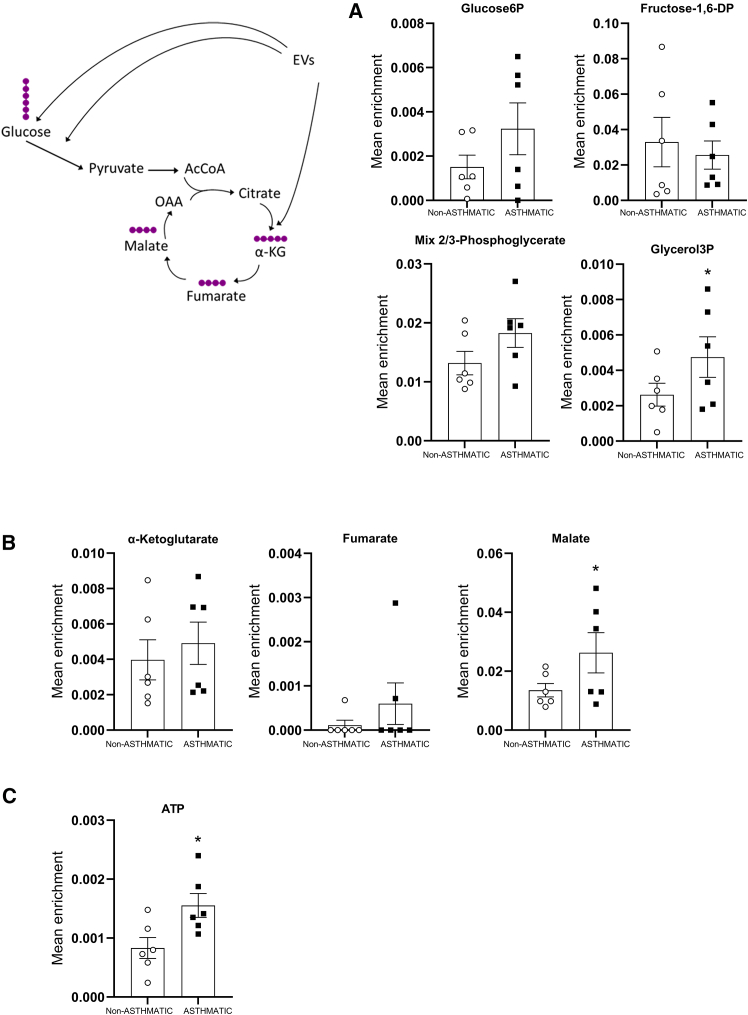
Bronchial epithelium consumption and metabolization of EVs Bronchial epithelium were treated with ^13^C enriched EVs from non-asthmatic (white circle, *n* = 6) and asthmatic (black square, *n* = 6) BSM cells before extraction and measurement of ^13^C-labeled metabolites using metabolomic analysis after 24 h. Incorporation of ^13^C was observed in glycolysis (A), Krebs’s cycle (B) and adenosine triphosphate (C).

In order to demonstrate that bronchial epithelium actively consumed and metabolized EVs contents, we isolated EVs-^13^ C from either non-asthmatic or asthmatic BSM cells cultured with a ^13^C-enriched cell culture medium. EVs-^13^ C were transferred to bronchial epithelium within the standard cell culture medium. Following metabolomic analysis, we were able to detect incorporation of ^13^C into metabolites, including those of glycolysis and Krebs’ cycle intermediates (Figures 4A and 4B). Due to a challenging experimental design, resulting in quantification of C^13^ label from EVs despite high glucose-C^12^ from the cell culture medium, we were able to observe significative results for C^13^ incorporation in glycerol-3P and malate. These results indicated active consumption followed by degradation and incorporation of C^13^ contained within EVs. Also, ^13^C incorporation was detected in adenosine nucleotides and especially in ATP (Figure 4C). Indeed, we observed a significant increased label of C^13^ into ATP from bronchial epithelium challenged with asthmatic BSM cells-derived EVs compared to that challenged with non-asthmatic BSM cells-derived EVs (Figure 4C).

### Asthmatic BSM cells-derived EVs alter the bronchial epithelium function and barrier defense capacities

**Figure 5:**
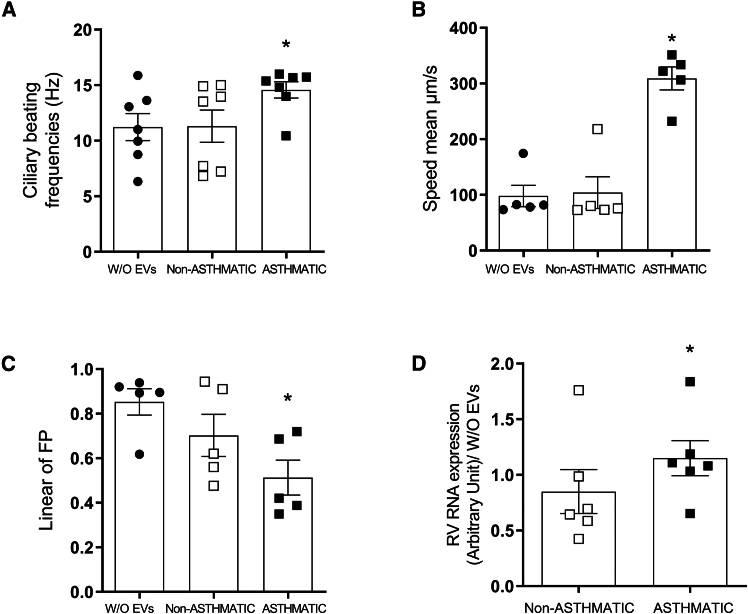
Asthmatic BSM cells derived EVs perturbated bronchial epithelium function (A) Ciliary beating frequency was measured using videomicroscopy in bronchial epithelium cultured without EVs (black circle, *n* = 7), non-asthmatic (white square, *n* = 7) or asthmatic (black square, *n* = 7) BSM cells-derived EVs during 6 h. After 6 h, efficiency of bronchial epithelium beating was assessed and analyzed using videomicroscopy. Mean speed (B) and linear of forward progression parameter (C) were measured in BE cultured without EVs (black circle, *n* = 5), non-asthmatic (white square, *n* = 5) or asthmatic (black square, *n* = 5) BSM cells-derived EVs. Bronchial epithelia were cultured with either non-asthmatic (white square, *n* = 6) or asthmatic (black square, *n* = 6) BSM cells derived EVs concomitantly with rhinovirus infection at MOI 0.1. Digital PCR was performed after 6 h after the infection (D). Data are presented as mean ± SEM. ∗*p* < 0.05.

Bronchial epithelium barrier defense capacities were analyzed through multiple experiments 6 h after the challenge with EVs. Indeed, using video microscopy associated to a high-speed camera, we recorded and measure the ciliary beating frequency. The ciliary beating frequency was significantly increased (14.57 ± 0.73 Hz) in the bronchial epithelium challenged with asthmatic BSM cells-derived EVs compared to that challenged with non-asthmatic BSM cells-derived EVs (11.31 ± 1.45 Hz) or untreated bronchial epithelium (11.2 ± 1.22 Hz) (Figure 5A) as represented by illustrative videos (Videos S1, S2, and S3). Using fluorescent latex beads at the top of the bronchial epithelium, we tracked the displacement and analyzed the speed mean and the linear forward progression index (Videos S4, S5, and S6). Both of these two indexes were significantly altered in bronchial epithelium challenged with asthmatic BSM cells-derived EVs compared to that challenged with non-asthmatic BSM cells-derived EVs or untreated bronchial epithelium (Figures 5B–5D). In particular, culturing the bronchial epithelium with asthmatic BSM cells-derived EVs increased by 3-fold the mean speed displacement of the beads (at 300 μm/s) (Figure 5B). By contrast, this major increase in the speed displacement was associated with a decrease in linear forward progression index leading to an anarchic displacement (Figure 5C). To complete the description of bronchial epithelium barrier function, we analyzed permeability and mucus composition using MUC5B and MUC5AC expression. Both properties were not significantly altered in bronchial epithelium challenged with asthmatic BSM cells-derived EVs compared to that challenged with non-asthmatic BSM cells-derived EVs or untreated bronchial epithelium (Figure S5).

Video S1. Ciliary beating frequency without EVs

Video S2. Ciliary beating frequency with non-asthmatic BSM cells-derived EVs

Video S3. Ciliary beating frequency with asthmatic BSM cells-derived EVs

Video S4. Beads tracking without EVs

Video S5. Beads tracking with non-asthmatic BSM cells-derived EVs

Video S6. Beads tracking with asthmatic BSM cells-derived EVs

In another set of experiments, bronchial epitheliums were cultured with EVs and infected with human rhinovirus RV16 at a multiplicity of infection (MOI) of 0.1, which emulated an *in vivo* infection of the bronchial epithelium.^40^ Bronchial epitheliums were collected 6h and 24h after infection. Prior to rhinovirus infection, we have analyzed the level of ICAM1 protein expression, the main receptor for RV-16, which was not altered between untreated bronchial epithelium and bronchial epithelium challenged with asthmatic BSM cells-derived EVs or non-asthmatic BSM cells-derived EVs (Figure S6A). The number of rhinovirus RNA particles detected by digital PCR (Figure 5D) was significantly increased 6 h post-infection in bronchial epithelium challenged with asthmatic BSM cells-derived EVs compared to that challenged with non-asthmatic BSM-derived EVs. Such a short time interval allowed the rhinovirus to enter the bronchial epithelial cells but did not allow its replication.^41^ We studied the inflammatory response following rhinovirus infection 6 h post-infection. Not surprisingly, we did not observed differences in specific rhinovirus associated interferon response such as IFNβ, IFNγ and IFNλ1-3 neither inflammatory epithelial secretion of alarmins such as TSLP or IL-33 (Figures S6B–6F). Thus, asthmatic BSM cells-derived EVs increased rhinovirus entry into the bronchial epithelium. Interestingly, ciliary beating frequency and efficiency of beating were not affected at 24 h after the EVs transfer (Figures S7A–S7C). Also, we did not observe any change in rhinovirus RNA expression at 24 h post-infection (Figure S7D).

## Discussion

Taken together, these results demonstrated that BSM cells from severe asthmatic patients produce more EVs, which in turn rewire the energetic metabolism of the bronchial epithelium, increasing ATP production and ciliary beating frequency but decreasing its efficiency. These major modifications disrupt the barrier function, leading to increased rhinovirus entry within the bronchial epithelium.

It is well admitted that bronchial epithelium could act on various cells such as the structural BSM cells. For instance, bronchial epithelium-derived factors such as epithelial growth factor,^42^^,^^43^ leukotrienes,^44^ or CXCL10^45^ induced both BSM cell proliferation and migration. By contrast, we previously showed a direct bottom-up effect of severe asthmatic BSM on bronchial epithelium through a CCL20/CCR6/Jnk/PKR/eIF2 signaling pathway, leading to increased rhinovirus replication.^20^ In the present study, we did not find any CCL20 within the BSM cells-derived EVs, and adding CCL20 on bronchial epithelial cells did not increase the level of ATP (Figure S8). We thus identified a new original bottom-up communication between BSM and bronchial epithelium which involve EVs leading to dramatic epithelial functional consequences essential for severe asthma trajectories.

Literature of EVs as intercellular communications mediators have already been described in lung diseases. Unfortunately, EVs mechanisms have been largely more studied in COPD rather than in asthma. Most of EVs provider’s cells are inflammatory cells such as macrophages, dendritic cells, eosinophils or neutrophils.^46^ Only few scientific articles describe the ability for structural cells (i.e., bronchial epithelial cells and fibroblasts) to produce EVs.^46^ Indeed, EVs are detected using their properties to express several markers such as ALIX and CD63 that only led to indirect or *ex vivo* detection of EVs.^46^ Pathological conditions were always associated with an increase level of EVs, found in patients’ bronchoalveolar lavage or blood serum, indicating that EVs quantity could be associated with the severity of the disease.^47^ In pathological context such as COPD, EVs were mostly associated with an increased inflammatory state. It is difficult to understand the real contribution for EVs in the development or the contribution of inflammation within the lung, however, COPD EVs transfer performed in naive mice could enhance the development of the disease indicating an effective transfer of EVs content and contribution of pathophysiological mechanism.^48^ In asthma, the contribution of EVs derived from immune and inflammatory cells has been previously demonstrated in the maintenance of asthma inflammation. Indeed, exosomes derived from mast cells have been implicated in communication with immune cells and activation of inflammatory pathways.^49^^,^^50^ Moreover, EVs derived from eosinophils, participated in the eosinophilic function and the maintenance of eosinophilic stimuli inflammation.^51^^,^^52^^,^^53^ T-cells could also generate EVs in order to activate mast cells.^54^^,^^55^ Nevertheless, only few articles have demonstrated the ability for structural cells to generate EVs. Kulshreshtha et al. have shown that bronchial epithelium, under IL-13 stimulation, were responsible for the emission of EVs that could attract macrophages.^56^ More recently, another article highlighted the ability of bronchial epithelial cells to produce EVs that participate in the proliferation, migration and activation of monocytes-derived dendritic cells.^57^ Fibroblastic cells have also been shown to produce EVs that could stimulate proliferation of epithelial cells through TGF-β2 signaling pathway.^58^ Hough et al., found that the concentration of EVs was increased in asthmatic bronchoalveolar lavage compared to that of healthy population.^34^ To the best of our knowledge, the present study is the first demonstrating the property for asthmatic BSM cells to generate EVs with several major functional epithelial consequences. Also, we cannot impute this increased production of EVs to an increased expression of EVs associated release protein, such as ESCRT family or CD63, since we did not identify or observe any modifications of protein expression between asthmatic and non-asthmatic BSM cells.

Indeed, we have shown that asthmatic BSM cells-derived EVs induced a proteome remodeling of bronchial epithelial cells. The modifications of key enzymes and proteins support the reorientation of glucose catabolism through glycolysis and mitochondrial degradation. EVs regulating metabolism of host cells was not described in asthma so far, but have been largely described in the field of cancer. Indeed, surrounding cells composing the tumor microenvironment have shown the ability to generate exosomes that could transfer metabolites to cancer cells. The transfer of specific metabolites inhibited the mitochondrial function and exacerbated the glycolysis pathway of cancer cells.^32^ Our study also demonstrated that EVs are consumed by bronchial epithelium and their contents served as energetic fuel. Indeed, using ^13^C-labelled glucose, pyruvate and glutamine, we were able to generate EVs enriched in ^13^C-metabolites. Epithelium challenged with asthmatic derived-BSM EVs were significantly altered metabolically with either an increased glycolysis and oxidative mitochondrial capacities. After stimulation of bronchial epithelium with ^13^C-EVs, ^13^C was detected in both glucose catabolism intermediates and Krebs’ cycle intermediates, indicating a large variety of energetic pathways of degradation. To the best of our knowledge, only one study, describing EVs from cancer associated cells,^32^ has performed such a clear demonstration of incorporation of EVs content into the different energetic pathways within the host cells.

In our study, we used non-asthmatic bronchial epithelium rather than asthmatic bronchial epithelium. Indeed, we strongly believe than there is an ascendant communication between BSM and bronchial epithelial cells. In a recent study, we showed that asthmatic BSM cells were responsible for increasing rhinovirus replication within bronchial epithelial cells.^20^ This major result was observed either in non-asthmatic and asthmatic bronchial epithelium, meaning that only the asthmatic BSM cells were sufficient for inducing their specific effect on rhinovirus response. Also, two additional studies reported that the rhinovirus level of replication between asthmatic and non-asthmatic BE was similar.^59^^,^^60^ Nevertheless, Ravi et al., showed that severe asthmatic bronchial epitheliums were metabolically different from non-severe and non-asthmatic epithelium, with a metabolic shift toward glycolysis and an impairment of oxidative phosphorylation.^61^ Conversely, previous studies have suggested that virus can reprogram host cell metabolic pathways.^62^^,^^63^ In the field of asthma, it is still unclear whether energetic modifications from severe asthmatic bronchial epithelium were the result of past rhinoviral infection. In this context, we performed all the experiments using non-asthmatic bronchial epithelium to clearly decipher the effect of asthmatic BSM-derived EVs.

In the present study, we highlighted the specific role for EVs derived from asthmatic BSM to modulate the host cell metabolism resulting in an increased rhinovirus infectivity. All the patients were classified as “severe” asthma. Indeed, non-severe asthmatic patients harbored a well-controlled asthma and likewise, no fiberoptic bronchoscopy could be performed on non-severe asthmatic patients. In this context, we cannot imagine using EVs for asthma therapies since the literature based on EVs therapeutics tend to only develop a potential role for EVs to deliver drugs in lung diseases.^64^ On the other hand, we believe this work confirms the implication of BSM in exacerbations and physiopathology of asthma and will appeal for new therapeutic development in asthma and more precisely therapeutic strategies to target the BSM remodeling in asthma.

### Limitations of the study

Several limitations have to be considered. First, specialists of the field of EVs and exosomes have contributed to an open discussion with an editorial in order to point out the right nomenclature in reporting EVs research.^65^ In this study, we paid specific attention to refer to them as EVs and not exosomes since the electronic microcopy have highlighted the standard size range for EVs after our experimental collection.

Second, we observed the biological effect of EVs on bronchial epithelial ciliary function at 6 h post-transfer only. We could hypothesize that if EVs were renewed every 6 h, effects on ciliary beating frequency, efficiency, and rhinovirus infection will be maintained since EVs’ metabolic substrates will feed bronchial epithelial cells continuously as it is happening *in vivo*.

Third, we only used human samples *in vitro*. Thus, we did not confirm our findings *in vivo* using an animal model of asthma. However, whereas animal models have been widely used in the literature, their results cannot be extrapolated systematically to human diseases. Indeed, there is no real animal model of severe asthma, particularly non-allergic severe asthma. Also, the use of rhinovirus RV16 is not possible in mice since its receptor, ICAM-1, is not expressed by murine bronchial epithelial cells.^66^ Moreover, most of the studies deciphering intercellular communication using EVs in asthma have been performed *in vitro* using immortalized cells or primary cell cultures from asthmatic patients.^67^

In conclusion, this study described a direct bottom-up effect of BSM cells-derived EVs from severe asthmatic patients on the energetic metabolism of bronchial epithelium, altering ciliary function and leading to increase rhinovirus entry.

## Resource availability

### Lead contact

Further information and requests for resources and reagents should be directed to and will be fulfilled by the lead contact, Pauline Esteves (pauline.esteves@u-bordeaux.fr).

### Materials availability

All unique/stable reagents generated in this study are available from the lead contact without restriction.

### Data and code availability

• All data reported in this paper will be shared by the lead contact upon request.
• Proteomic datasets were downloaded PRIDE under accession number PXD055968, PXD055949, PXD059603.
• This paper does not report original code.

## Acknowledgments

We thank the staffs of both the pathology and surgery departments (both from the University Hospital of Bordeaux), Isabelle Goasdoue, Natacha Robert, Marine Douillet, and Arnaud Rulie from the clinical investigation center, for technical assistance. We thank Nancy Geoffre and Cyrielle Clément for lipidomic analysis.

Sources of support: The project was funded by the “Fondation de l’Université de Bordeaux (Fonds pour les maladies chroniques nécessitant une assistance médico-technique FGLMR/AVAD)”, “10.13039/501100002915Fondation pour la Recherche Médicale” (DEQ20170336706), “10.13039/501100001665Agence Nationale de la Recherche” (ANR, VIRCHILLD ANR-21-CE14-0074) and an unrestricted grant from 10.13039/100019717AstraZeneca.

## Author contributions

E.C., A.C., and P.E. performed the vast majority of the *in vitro* experiments and wrote the first draft of the manuscript. F.B., G.C., E.E., C.B., M.C., and O.O. technically assisted with the *in vitro* experiments. M.T., P.O.G., and P.B. conducted patient recruitment. J.-W.D. performed the proteomics experiments. T.L.-L. performed the digital PCR experiments. R.D. developed the MATHLAB program for ciliary beating frequency analysis. S.L. performed the electronic microscopy. N.L.-L. and F.B. performed the metabolomics experiments. R.M., T.T., and P.B. helped to design the study and edited the manuscript. T.T., P.B., and P.E. conceived the project, designed and supervised the study, analyzed the data, and edited the manuscript.

## Declaration of interests

The authors declare no competing interests.

## STAR★Methods

### Key resources table

**Table:** 

REAGENT or RESOURCE	SOURCE	IDENTIFIER
**Antibodies**		

anti-ALIX	abcam	Cat# ab275377; AB_3644262
anti-CD63	abcam	Cat# ab134045; AB_2800495
anti-LDHA	Cell Signaling	Cat# 3582; AB_2066887
anti-LDHB	Cell Signaling	Cat# 56298; AB_3678824
anti-CD63 PE	Invitrogen	Cat# MA1-19650; AB_1073281
anti-ICAM1	Cell Signaling	Cat# 4915; AB_2280018

**Bacterial and virus strains**		

Human rhinovirus 16	ATCC	VR-283

**Chemicals, peptides, and recombinant proteins**		

Human CD63 isolation	Invitrogen	10606D
Latex beads luorescent red	Sigma-Aldrich	L3030

**Critical commercial assays**		

CellTiter-Glo ATP assay	Promega	G9241
Human MUC5AC ELISA kit	Bio-Techne	NBP2-76703
Human MUC5B ELISA kit	Bio-Techne	NBP2-76705
Human IFN-beta ELISA	Bio-Techne	DY814-05
Human IFN-Lambda1/3 ELISA	Bio-Techne	DY1598B
Human IFN-gamma ELISA	Bio-Techne	DY285B
Human TSLP ELISA	Bio-Techne	DY1398
Human IL-33 ELISA	Bio-Techne	DY3625B
RNeasy Mini Kit	QIAGEN	74104
QX200 ddPCR EvaGreen Supermix	BioRad	186-4033

**Deposited data**		

EVs proteomic analysis	PRIDE	PXD055968
Proteomic analysis of bronchial epithelium challenged with bronchial smooth muscle extracellular vesicles (24h)	PRIDE	PXD055949
Proteomic analysis of bronchial epithelium challenged with bronchial smooth muscle extracellular vesicles (6h)	PRIDE	PXD059603

**Oligonucleotides**		

RV primers	F:5’-AGCCTGCGTGGCKGCC-3’ R:5’-GAAACACGGACACCCAAAGTAGT-3’	

### Experimental model and subject details

43 subjects were recruited for this study and subdivided into two groups: severe asthmatic patients and non-asthmatic subjects. A total of 15 patients with severe asthma were prospectively recruited at the University Hospital of Bordeaux, France, from the COBRA cohort (COhort of BRonchial obstruction and Asthma). The severe asthma diagnosis was established by clinical physicists according to ATS/ERS criteria and GINA recommendations.^68^ Non-asthmatic subjects were recruited after surgical resection. A total of 28 non-asthmatic subjects were then prospectively recruited from the University Hospital of Bordeaux. Bronchial specimens from all subjects were obtained by either fiberoptic bronchoscopy or lobectomy in macroscopically normal area, as previously described.^42^ All subjects gave their written informed consent to participate in the study after the nature of the procedure had been fully explained.

### Method details

#### Cell culture

Bronchial smooth muscle (BSM) cells were obtained from patients’ biopsies and bronchi dissected out from lobectomy, as described previously.^4^ Briefly, BSM cell culture was performed in DMEM 25mM Glucose (Gibco, Thermo Fisher Scientific), supplemented with 10% FBS (Gibco) and penicillin-streptomycin-Amphotericin B 1X (100X; Gibco) and essential amino acids 1X (100X; Sigma-Aldrich). The smooth muscle phenotype was confirmed by immunocytochemistry using the double staining of smooth muscle alpha-actin and calponin. Bronchial epithelial cell culture was established from bronchial brushings patients’ biopsies and bronchi dissected out from lobectomy, as previously described in Trian et al.,^44^ using PneumaCult medium (Stemcell).

#### Extracellular vesicles purification

Extracellular vesicles (EVs) were collected from BSM cells medium. BSM cells were culture at maximal confluency during 72h. The cell culture medium was collected and centrifugated through several successive centrifugations: 300 g during 10 min, 2000 g during 20 min and 10 000 g during 10 minutes. Cell culture medium was then transferred for ultracentrifugation using rotor type 70 Ti (Beckman Coulter) into dedicated tubes (Beckman Coulter) for 100 000 g during 1 hour and 30 minutes. The cell culture medium was removed before adding PBS and another ultracentrifugation at 100 000 g for 1 hour and 30 minutes. All of the centrifugation and ultracentrifugation were performed at 4°C. EVs were resuspended in PBS and stored at -80°C. A working concentration of 0.2 mg/ml was chosen since the range for EVs utilization in cell cultures was reported in the literature from 0.1-0.4 mg/ml.^32^^,^^69^^,^^70^

#### Western-blotting

Total cells and biopsies lysis were performed using a RIPA lysis buffer (Sigma-Aldrich). Total cellular extracts were loaded onto a 4-20 % SDS-PAGE gel (Bio-Rad), transferred onto nitrocellulose membrane. Different commercial antibodies were used directed against ALIX (Abcam), CD63 (Abcam), LDHA (Cell Signaling) and LDHB (Cell Signaling). HRP-coupled secondary antibodies were used for revelation using ChemiDoc imaging instrument (Bio-Rad). Protein expressions were normalized using total loading protein intensity (Stain-free system Bio-Rad).

#### Extracellular vesicles numeration by flow cytometry

Purified EVs were detected and counted using Dynabeads magnetic beads labelled for CD63 (Fisher Scientific, Waltham, MA) coupled with staining using CD63-PE (Fisher Scientific, Waltham, MA). Dynabeads-CD63 were relatively large (*i.e.* 4.5 μm in diameter) which led us to clearly defined FFC/SSC and gating of EVs population. FACS data were acquired using a Canto II 4-Blue 2-Violet 2-Red laser configuration (BD Biosciences). Flow cytometry analyses were performed using Diva 8 (BD Biosciences).

#### Electronic microscopy

Imaging was performed on the Bordeaux Imaging Center, member of the FranceBioImaging national infrastructure (ANR-10-INBS-04). EVs were concentrated and resuspended in an equal volume of 4% PFA in PBS (2% PFA final). 20 μL drops of the resuspended exosomes were then absorbed for 20 min at RT on hydrophilic carbon coated grids. After PBS rinses, grids were placed on drops of 1% glutaraldehyde in PBS for 5 min. Several washes were done with distilled water, and grids were then transferred on a filtered drop of pH7 uranyl-oxalate solution, for 5 min in the dark. Grids were then transferred in drops of filtered 4% aqueous uranyl acetate/2,3M methylcellulose (1V for 9V) on a petri dish covered with parafilm on ice for 10 min in the dark. Grids were then removed, one at time, with a stainless-steel loop, and excess fluid were blotted by gently touching the edge of the loop with a Whatman No. 1 filter paper. Grids were air-dried while still on the loop, torn off and stored in a grid storage box. Grids were examined with a Transmission Electron Microscope (H7650, Hitachi, Tokyo, Japan) at 80kV in High Contrast Mode, equipped with a CCD camera Orius 11MPx (Gatan, Paris (France). The TEM pictures are done using Digital Micrograph software (Gatan company).

#### Label-free quantitative proteomics

Cells lysis were processed using RIPA buffer. Each lysate was centrifuged and the supernatant was used for the proteomic analysis. The proteins from exosomes were desalted and digested in an SDS-PAGE gel method^20^ and the proteins from epithelium using the Single-pot, solid-phase-enhanced sample preparation (SP3) method.^71^ NanoLC-MS/MS analysis were performed for exosomes using Ultimate 3000 RSLC Nano-UPHLC system (Thermo Scientific, USA) coupled to a nanospray Orbitrap Fusion™ Lumos™ Tribrid™ (Thermo Fisher Scientific, California, USA, see parameters:^20^) and for epithelium: Vanquish Neo UHPLC System (Thermo Scientific) associated to Orbitrap Exploris™ 480 (see below parameters). The peptide extracts were loaded onto a 5 mm × 300 μm ID PepMap Neo Trap Cartridge (C18, 5 μm particle size, 100 Å pore size, Thermo Scientific) and separated on an analytical column (25 cm × 75 μm ID, 1.7 μm, C18 beads, Ionopticks) at a flow rate of 300 nL/min at 50°C using a multistep gradient of 3–25% mobile phase B (80% MeCN in 0.1% formic acid) for 45 minutes and 25–35% B for 15 minutes, 35–95% B for 1 minute and an 11-minute wash at 99% B. The mass spectrometer operated in positive ion mode at a 1.4 kV needle voltage, and data were acquired using Xcalibur 4.5 software in a data-dependent mode. MS scans (m/z 375–1500) were recorded at a resolution of R = 120000 (@ m/z 200), a standard AGC target, and an injection time in automatic mode, followed by a top speed duty cycle of up to 1 second for MS/MS acquisition. Precursor ions (2–6 charge states) were isolated in the quadrupole with a mass window of 2 Th and fragmented with HCD @ 30% normalized collision energy. MS/MS data were acquired with a resolution of R = 15000 (@m/z 200), a standard AGC target, and a maximum injection time in automatic mode. Selected precursors were excluded for 45 seconds.

Protein identification and label-free quantification (LFQ) were done in Proteome Discoverer 3.0. The CHIMERYS node using the prediction model inferys_2.1 fragmentations was used to identify proteins in batch mode by searching against the UniProt *Homo sapiens* database (82408 entries, released September 2023). Two missed enzyme cleavages were allowed for trypsin. Peptide lengths of 7–30 amino acids, a maximum of 3 modifications, charges of 2–4, and 20 ppm for fragment mass tolerance were set. Oxidation (M) and carbamidomethyl (C) were respectively searched as dynamic and static modifications by the CHIMERYS software. Peptide validation was performed using the Percolator algorithm and only “high confidence” peptides were retained corresponding to a 1% false discovery rate at the peptide level. Minora feature detector node (LFQ) was used along with the feature mapper and precursor ions quantifier. The normalization parameters were selected as follows: (1) Unique peptides, (2) Precursor abundance based on intensity, (3) Normalization mode: total peptide amount, (4) Protein abundance calculation: summed abundances, (5) Protein ratio calculation: pairwise ratio based and (6) Missing values were replaced with random values sampled from the lower 5% of detected values. Quantitative data were considered for master proteins and quantified by a minimum of 2 unique peptides. The mass spectrometry proteomics data have been deposited to the ProteomeXchange Consortium via the PRIDE ^72^ partner repository with the dataset identifiers PXD055968 and PXD055949. Proteins were clustered according to their functions by using the Kyoto Encyclopedia of genes and genome analysis in the search tool for retrieval of interaction between genes and proteins (STRING) database. A more global analysis of the data was performed using Ingenuity Pathway Analysis (IPA; Qiagen). We used the ‘Core Analysis’ package to identify relationships, mechanisms, functions, and pathways relevant to a dataset. We also used the ‘regulators’ package to identify predicted regulators of the proteomic changes. Comparative analyses were also performed with IPA using the ‘Comparative Analysis’ module.

#### Cell culture substrate concentration measurement

Both glucose and lactate concentrations were measured in the cell culture medium of BE co-cultured with BSM cells-derived EVs using nuclear magnetic resonance (NMR) spectrometry performed at Metatoul (Metatoul, Toulouse, France). NMR samples were prepared by mixing 180 μL of supernatant with 20 μL of an internal standard solution containing 10 mM of deuterated trimethylsilylpropanoic acid (TSP-d4) in D_2_O. 1D ^1^H NMR spectra were acquired on an Avance Neo 800 MHz NMR spectrometer equipped with a 5 mm CQPCI Z-Gradient cryoprobe (Bruker Biospin, Wissembourg). Quantitative NMR analysis was performed at 280 K, using a projectpr1d pulse sequence with 32 scans, 16 dummy scans, 128k data points, a spectral width of 20 ppm and an interscan delay of 11,06 s. NMR spectra were processed using TopSpin 4.1.4 software (Bruker, Germany). First, a Fourier transform was applied with an exponential line broadening of 0.3 Hz and a zero-filling of 128K points, then the phase and baseline of each spectrum were corrected. Absolute quantitation of metabolites was obtained using the internal standard TSP-d4.

#### Cellular oxygen consumption rate

Cellular oxygen consumption rate (OCR) was measured on intact cells at 37°C in a 2 mL thermostatically monitored chamber (2.5 x 10^5^ cells/ ml / run) using an Oroboros O2k instrument (Oroboros Instruments). BE inserts were gently detached from their support and left onto the chamber. One BE insert was used per chamber. High-resolution respirometry was determined under routine condition in DMEM 5mM of glucose without fetal bovine serum addition. The cellular non-mitochondrial respiration was obtained after inhibition of the respiratory chain using a mix of antimycin A and potassium cyanide respectively 5 μM and 2.5 mM.

#### Cellular ATP synthesis

The steady-state ATP content was measured by bioluminescence using the CellTiter Glo kit (Promega), following manufacturer’s recommendations.

#### Metabolites and lipids analysis by IC/HRMS

Extraction and quenching of metabolites from EVs were performed using a specific protocol from Metatoul (Metatoul, Toulouse, France) as previously described by Heuillet M. et al. Metabolites were analyzed by ionic-exchange chromatography coupled with high resolution mass spectrometry (IC/HRMS) using the method described^73^). For ^13^C enrichment EVs, bronchial smooth muscle cells were cultured in DMEM no glucose supplemented with 25 mM of ^13^C-glucose, 2 mM ^13^C-sodium pyruvate and 2 mM ^13^C-glutamine.

Lipids corresponding to (nature and quantity of samples) were extracted according to a double extraction. Samples are quenched with 4 ml of ACN/MeOH/water (2:2:1; 125mM formic acid). Internal standards (45uL IDMS, 100uL LN standard and 40uL phospolipid standard) are added. SThe samples are mixed and stored at -20 degrees for 30min. 1mL of water and 2.5mL of CH2Cl2 are added to the samples and mixed. Then samples are centrifuged 6min at 2500 rpm. The aqueous phase is recovered for polar metabolite analysis. The organic phase is dried and concentrated in 20 μL of ethyl acetate for neutral lipids analysis or 50 μL pf methanol for phospholipids analysis. Lipid extracts were analyzed using an Agilent 1290 UPLC system coupled to a G6460 triple quadripole mass spectrometer (Agilent Technologies) and using MassHunter software for data acquisition and analysis. A Kinetex HILIC column (Phenomenex, 50 x 4.6 mm, 2.6 μm) was used for LC separations. Analyses were performed in Selected Reaction Monitoring detection mode (SRM) using nitrogen as collision gas. Ion optics and collision energy were optimized for each lipid class. Finally, peak detection, integration and quantitative analysis were done using MassHunter Quantitative analysis software (Agilent Technologies).

#### Rhinovirus infection

The human rhinovirus RV-16 was purchased from ATCC. BE cells were infected during 1 hour with 0.1 MOI RV. Such a low MOI allowed us to observe virus replication without any leaking of rhinovirus particles in either the co-culture medium. Indeed, no rhinovirus RNA particle was found in co-culture medium using digital PCR.^20^ Experiments were performed 6h and 24h post-infection.

#### RNA extraction and digital PCR

Total RNA was extracted from cells using a Qiagen RNeasy kit (Qiagen) by following the manufacturer’s recommendations. PCRs were prepared with the required reagent, QX200 ddPCR EvaGreen Supermix (Bio-Rad), at a final concentration of 150 nM for each primer in a final volume of 22 μl. Each reaction was performed using 4 ng of cDNA.

#### Ciliary beating frequency

Ciliary beating frequency of BE were measured using videomicroscopy Leica DMi8 (Leica Microsystems) coupled to a high-speed camera sCMOS Flash 4.0 camera (Hamamatsu) available at The Bordeaux Imaging Center. The illumination system used was Cool LED PE-4000 (CoolLED) associated to a HCX PL Fluotar L 40X dry 0.6 NA PH2 objective. BE were placed in a 37°C chamber using an incubator box equipped with a gas heating system (Pecon GmbH). The acquisitions were carried out during 2 seconds at a frequency of 1000 images/sec so a total of 2001 images, on a defined zone (100x100 pixels) using MetaMorph software (Molecular Devices). Using MATHLAB program, the images were analyzed after application of the Fourier transform to determine the most represented beat frequency.

#### Ciliary beating efficiency

Ciliary efficiency was analyzed through the movement of fluorescent beads at the apical side of BE using videomicroscopy DMI8 (Leica). BE were rinsed three times using PBS in order to remove a maximum of mucus. Fluorescent latex beads were added to the top of BE (Sigma-Aldrich). A mix of 10μL of beads was diluted in 990 μL of DMEM complemented with 2% FBS solution (Fisher Scientific). Mix of fluorescent beads was applied to the apical side of BE for 2 min at 37°. Tracking of fluorescent latex beads at the cell surface of the BE was performed thanks to an ImageJ plug-in, TrackMate. Two key parameters were extracted and analyzed: mean speed and linearity of forward progression. The mean speed, related to velocity was computed as the displacement from one frame to the next, divided by the time interval. The linearity of forward progression was defined as the ratio between the mean straight-line speed and the track mean speed.

#### Transepithelial resistance (TEER)

BE permeability was obtained by measuring the transepithelial resistance. The device allows the injection of current through a pair of electrodes. One of the electrodes will be positioned at the apical pole of BE while the second electrode will be placed at the basal pole. The electrical conductance, being the capacity of the cell layer to allow a specific amount of electric current to pass, will be obtained by subjecting the BE to a potential difference between the apical and basal poles. The TEER measurement will be carried out for 48 hours.

#### MUC5AC and MUC5B expression

Mucus composition was appreciated using ELISA (Enzyme-Linked Immuno-Sorbent Assay) by measuring the expression of MUC5AC and MUC5B mucins. Washes of BE using 300 μl of PBS were processed and immediately stored and freezed at -80°C for optimal preservation. MUC5AC and MUC5B mucins (human MUC5B and human MUC5AC, Bio-Techne). ELISA were performed using non-diluted BE washes and measured at 450 nm using the SPECTROstar device and the associated SPECTROstar Nano MARS software.

#### Interferon and alarmins expression

Interferon response (IFN β, IFN γ and IFN λ1-3) and alarmins (TSLP and IL-33) were appreciated using ELISA (Enzyme-Linked Immuno-Sorbent Assay). BE cell culture media were immediately stored and freezed at -80°C for optimal preservation. ELISA for interferons and alarmins (human IFN β, IFN γ, IFN λ1-3, TSLP and IL-33, Bio-Techne) were performed using non-diluted cell culture medium and measured at 450 nm using the SPECTROstar device and the associated SPECTROstar Nano MARS software.

### Quantification and statistical analysis

Except for the proteomics analyses, all the statistical analyses were performed with Prism 6 software (GraphPad). Values are presented as the mean ± SEM. Statistical significance, which was defined as *P* < 0.05, was determined with Student’s *t* test, analysis of variance, Bonferroni’s test and Chi’s square test.
