# Supplementary material for: Bronchial smooth muscle extracellular vesicles interfere with bronchial epithelium metabolism and function in asthma
Source: iScience. 2025 Apr 29;28(6):112546. doi: 10.1016/j.isci.2025.112546 (PMC12141081; doi:10.1016/j.isci.2025.112546)
Supplement: Document S1. Figures S1–S8 and Tables S1 and S2 [file mmc1.pdf]

iScience, Volume *28*

Supplemental information

Bronchial smooth muscle extracellular vesicles interfere with bronchial epithelium metabolism and function in asthma

Elisa Celle, Amine Chahin, Fabien Beaufils, Guillaume Cardouat, Edmée Eyraud, Clément Bouchet, Marilyne Campagnac, Olga Ousova, Hugues Begueret, Matthieu Thumerel, Rémi Dubois, Jean-William Dupuy, Thierry Leste-Lasserre, Sabrina Lacomme, Nina Lager-Lachaud, Floriant Bellvert, Roger Marthan, Pierre-Olivier Girodet, Patrick Berger, Thomas Trian, and Pauline Esteves

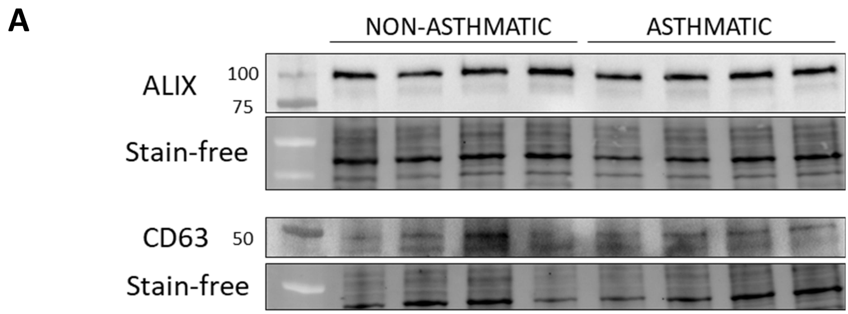

Supplemental figure 1

A

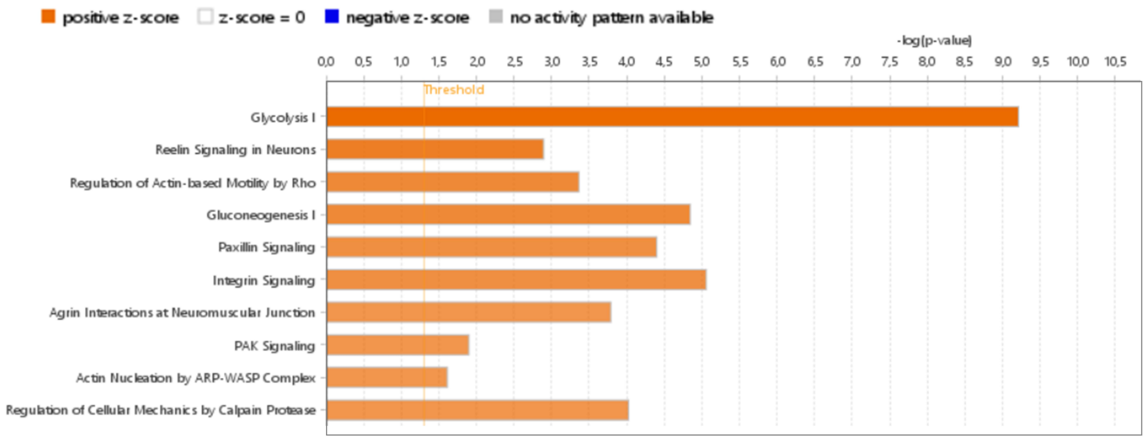

Supplemental figure 2

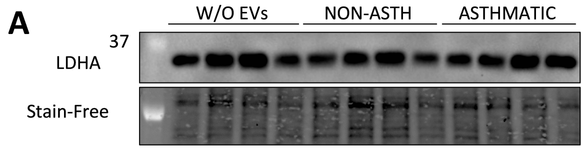

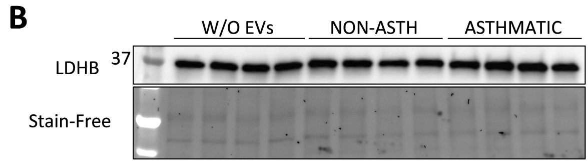

Supplemental figure 3

A

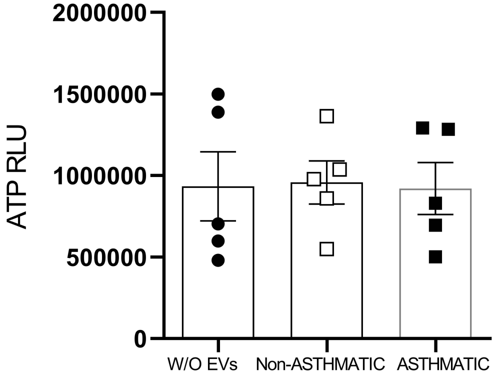

Supplemental figure 4

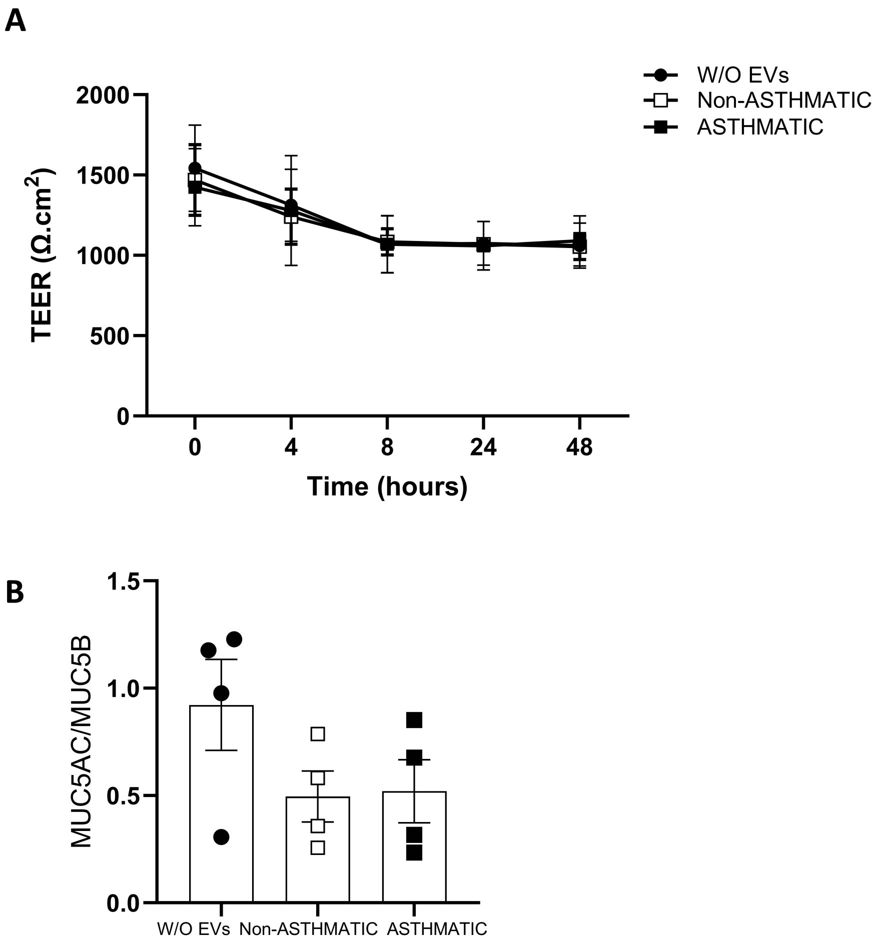

Supplemental figure 5

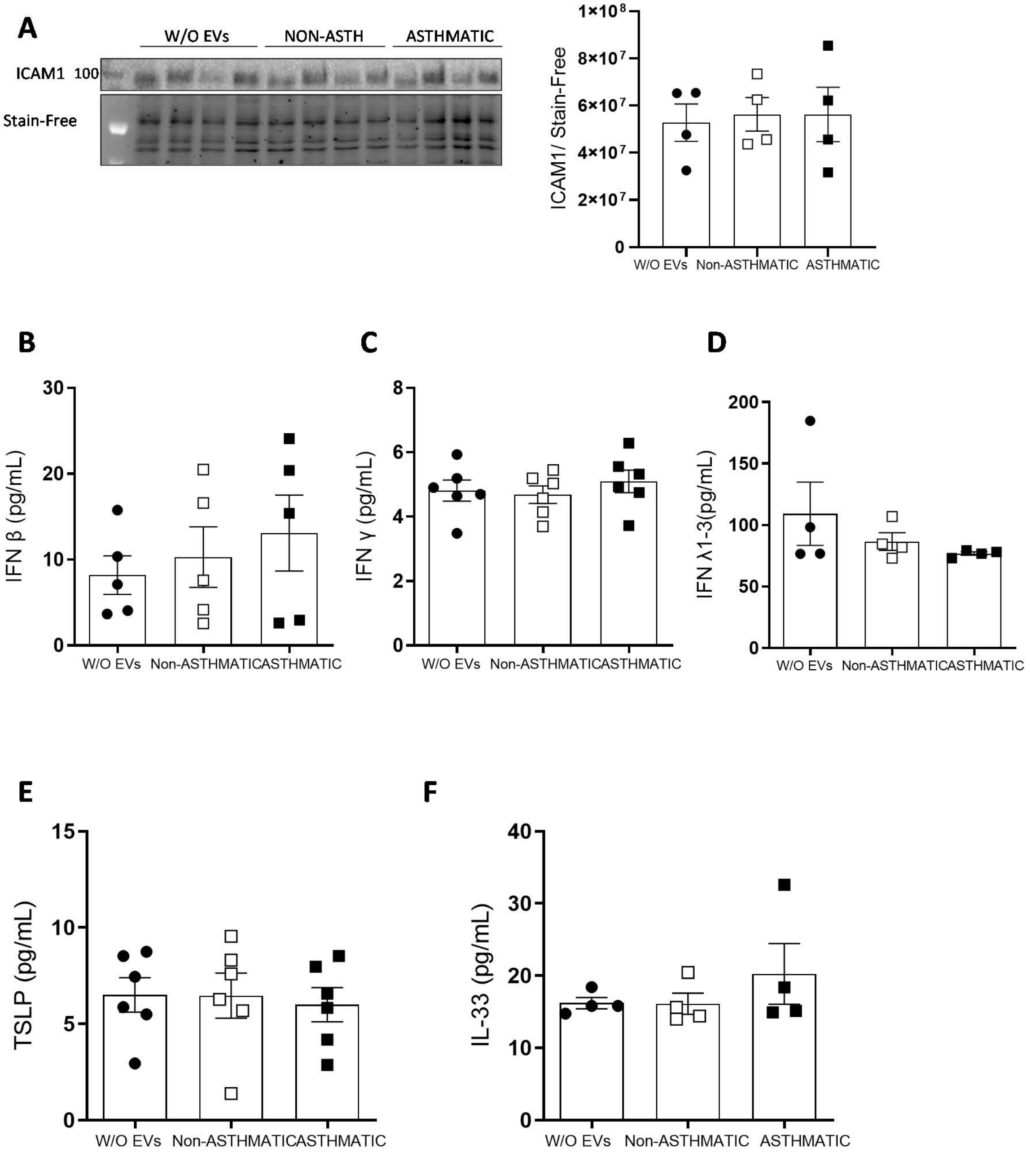

Supplemental figure 6

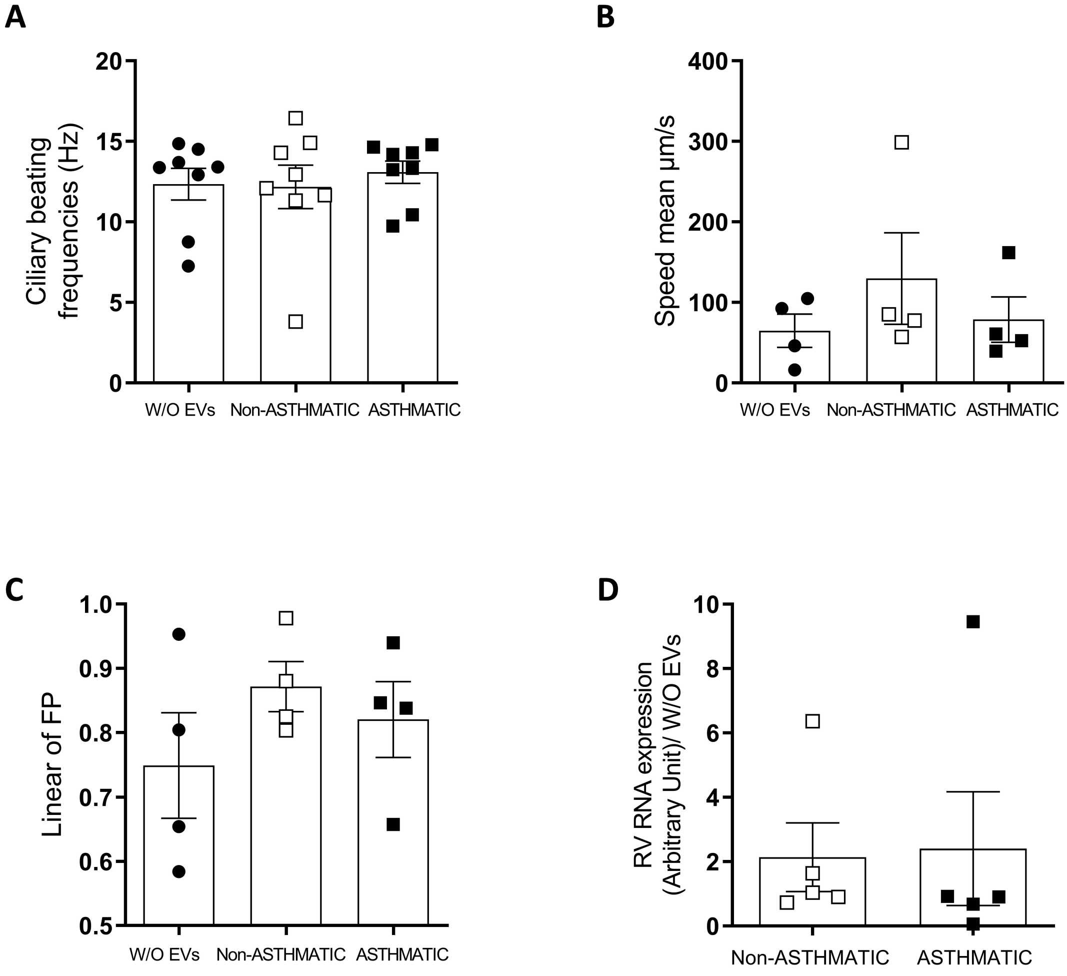

Supplemental figure 7

A

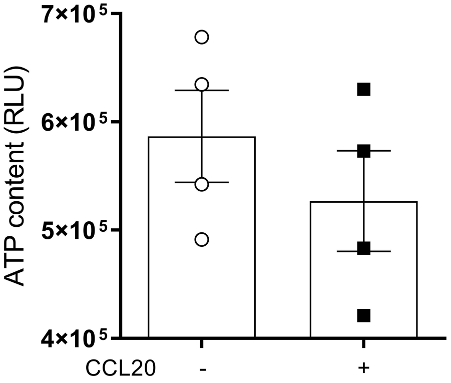

Supplemental figure 8

Supplemental figure 1: ALIX and CD63 protein expression in BSM cells
(A) ALIX and CD63 protein expression in non-asthmatic and BSM cells using Western-blotting. Stain-free gel technology was used for loading control expression.

Supplemental figure 2
(A) Proteome comparison between non-asthmatic and asthmatic BSM cells-derived EVs (n=3) was performed using mass spectrometry analysis associated with Ingenuity Pathway Analysis (IPA) from the raw proteomic data. The proteins with a different expression were organized based on pre-defined categories suggested by IPA. Then, the proteins with different expression were assigned to these IPA categories based on the IPA database. A top 10 categories were ranked according to their z-score and frequency of identification in the proteome [-log (p value)].

Supplemental figure 3
(A) Representative images of LDHA and **(B)** LDHB Western-blotting.

Supplemental figure 4
(A) Steady-state ATP content was measured in bronchial epithelium cultured without EVs (black circle, n=5), non-asthmatic (white square, n=5) and asthmatic (black square, n=5) BSM cells-derived EVs using a luminescence assay after 24 hours of EVs incubation. Data are presented as mean ± SEM. * $p < 0.05$.

Supplemental figure 5: Bronchial epithelium permeability and mucus quality
(A) Transepithelial resistance was measured in order to address bronchial epithelium permeability during 48 hours between bronchial epithelium cultured without EVs (black circle, n=4), non-asthmatic (white square, n=4) or asthmatic (black square, n=4) BSM cells-derived EVs. **(B)** Mucins MUC5AC and MUC5B were measured in mucus from bronchial epithelium cultured without EVs (black circle, n=4), non-asthmatic (white square, n=4) or asthmatic (black square, n=4) BSM cells-derived EVs during 6 hours.

Supplemental figure 6: ICAM1 expression, interferons and alarmins response
(A) ICAM1 protein expression in bronchial epithelium cultured without EVs (black circle, n=4), non-asthmatic (white square, n=4) or asthmatic (black square, n=4) BSM cells-derived EVs using Western-blotting. Stain-free gel technology was used for loading control expression. **(B-D)** Interferon response such as IFN β (n=5), IFN γ (n=6) and IFN λ1-3 (n=4) and alarmins **(E)** TSLP (n=6) and **(F)** IL-33 (n=4) were measured using ELISA in cell culture media from bronchial epithelium cultured without EVs (black circle), non-asthmatic (white square) or asthmatic (black square) BSM cells-derived EVs during 6 hours.

Supplemental figure 7: Bronchial epithelium function
(A) Ciliary beating frequency was measured using videomicroscopy in bronchial epithelium 24 hours after incubation without EVs (black circle, n=8), with non-asthmatic (white square, n=8) or asthmatic (black square, n=8) BSM cells-derived EVs. Efficiency of bronchial epithelium beating was assessed and analyzed using videomicroscopy. Mean speed **(B)** and linear of forward progression **(C)** parameters were measured in bronchial epithelium cultured without EVs (black circle, n=4), non-asthmatic (white square, n=4) or asthmatic (black square, n=4) BSM cells-derived EVs. Bronchial epithelium were cultured with either non-asthmatic (white square, n=5) or asthmatic (black square, n=5) BSM cells derived EVs concomitantly with rhinovirus infection at MOI 0.1. Digital PCR was performed after 24 hours after infection **(D)**. Data are presented as mean ± SEM. * $p < 0.05$.

Supplemental figure 8
(A) Steady-state ATP content was measured in untreated bronchial epithelium (white circle, n=4) or CCL20 treated bronchial epithelium during 24 hours (black square, n=4) using a luminescence assay. Data are presented as mean ± SEM. * $p < 0.05$.

Supplemental table 1

Ingenuity Canonical Pathways	-log(p-value)	Ratio	z-score	Molecules
Glycolysis I	9,21E00	2,96E-01	2,828	ALDOA,ENO1,GAPDH,PFKL,PGAM2,PGK1,PKM,TPI1
Reelin Signaling in Neurons	2,9E00	5,07E-02	2,449	APOE,APP,CDH2,CFL1,ITGA5,ITGB1,RAP1B
Gluconeogenesis I	4,85E00	1,85E-01	2,236	ALDOA,ENO1,GAPDH,PGAM2,PGK1
Regulation of Actin-based Motility by Rho	3,36E00	6,09E-02	2,236	ACTB,CFL1,GSN,ITGA2,ITGA5,ITGB1,PFN1
Paxillin Signaling	4,4E00	7,48E-02	2,121	ACTB,ACTN1,ITGA2,ITGA5,ITGB1,RAP1B,TLN1,VCL
Integrin Signaling	5,06E00	5,66E-02	2,111	ACTB,ACTN1,ARF3,GSN,ILK,ITGA2,ITGA5,ITGB1,PFN1,RAP1B,TLN1,VCL
Regulation of Cellular Mechanics by Calpain Protease	4,02E00	7,78E-02	2,000	ACTN1,ITGA2,ITGA5,ITGB1,RAP1B,TLN1,VCL
Agrin Interactions at Neuromuscular Junction	3,79E00	8,7E-02	2,000	ACTB,AGRN,ITGB1,LAMB1,LAMC1,RAP1B
VEGF Signaling	2,2E00	5,05E-02	2,000	ACTB,ACTN1,RAP1B,VCL,YWHAE
PAK Signaling	1,9E00	4,27E-02	2,000	CFL1,ITGA2,ITGA5,ITGB1,RAP1B
Actin Nucleation by ARP-WASP Complex	1,61E00	4,3E-02	2,000	ITGA2,ITGA5,ITGB1,RAP1B
Regulation of eIF4 and p70S6K Signaling	1,2E00	2,76E-02	2,000	EIF4A2,ITGA2,ITGA5,ITGB1,RAP1B
IL-6 Signaling	1,18E00	3,1E-02	2,000	A2M,COL1A1,HSPB1,RAP1B
HGF Signaling	1,15E00	3,03E-02	2,000	ITGA2,ITGA5,ITGB1,RAP1B
Endocannabinoid Cancer Inhibition Pathway	1,02E00	2,72E-02	2,000	CASP14,MMP2,PRKAR2A,VIM
Neuroinflammation Signaling Pathway	2,88E-01	1,26E-02	2,000	APP,HLA-B,MFGE8,MMP3
TEC Kinase Signaling	0	8,65E-03	2,000	ACTB,GNB1,ITGA2,ITGA5,ITGB1
T Cell Receptor Signaling	0	8,12E-03	2,000	CALML5,HLA-B,ITGA2,ITGB1,RAP1B
Synaptogenesis Signaling Pathway	4,61E00	4,44E-02	1,941	APOE,CALML5,CDH2,CFL1,COMP,HSPA8,LRP1,NAP1L1,PRKAR2A,RAP1B,THBS1,THBS2,THBS4,TLN1
ERK/MAPK Signaling	3,61E00	4,65E-02	1,890	HSPB1,ITGA2,ITGA5,ITGB1,PRKAR2A,RAP1B,TLN1,YWHAG,YWHAQ,YWHAZ
Ephrin Receptor Signaling	1,99E00	3,47E-02	1,890	CFL1,GNB1,ITGA2,ITGA5,ITGB1,RAP1B,SDCBP
Phospholipase C Signaling	0	7,14E-03	1,890	AHNAK,CALML5,GNB1,ITGA2,ITGA5,ITGB1,PEBP1,RAP1B
Actin Cytoskeleton Signaling	7,43E00	6,56E-02	1,807	ACTB,ACTN1,CFL1,F2,FLNA,FN1,GSN,ITGA2,ITGA5,ITGB1,MSN,MYH9,PFN1,RAP1B,TLN1,VCL
Wound Healing Signaling Pathway	1,54E01	9,92E-02	1,800	COL12A1,COL15A1,COL18A1,COL1A1,COL1A2,COL3A1,COL5A1,COL6A1,COL6A2,COL6A3,F2,FN1,ITGB1,KRT16,KRT17,KRT6A,KRT6B,KRT71,LAMA5,LAMB1,LAMC1,MMP1,MST1,RAP1B,VIM
Intrinsic Prothrombin Activation Pathway	8,91E00	2,14E-01	1,667	COL18A1,COL1A1,COL1A2,COL3A1,F10,F2,F5,FGB,SERPINC1
Cardiac Hypertrophy Signaling (Enhanced)	7,18E-01	1,66E-02	1,667	CALML5,FGFR1,GNB1,HSPB1,ITGA2,ITGA5,ITGB1,PRKAR2A,RAP1B
Osteoarthritis Pathway	6,83E00	6,36E-02	1,508	ANXA2,ANXA5,CASP14,DCN,FGFR1,FN1,GREM1,HTRA1,ITGA2,ITGA5,ITGB1,LRP1,MMP1,MMP3,RBP4
FAK Signaling	0	1,06E-02	1,508	COL18A1,COL1A1,COL1A2,COL3A1,ITGA2,ITGA5,ITGB1,MMP2,RAP1B,SDCBP,SPARCL1
Hepatic Fibrosis Signaling Pathway	4,9E00	4,02E-02	1,500	CALML5,COL18A1,COL1A1,COL1A2,COL3A1,FGFR1,FTH1,ITGA2,ITGA5,ITGB1,LRP1,MMP1,PRKAR2A,RAP1B,SERPINE1,TFRC,TIMP1
Adrenomedullin signaling pathway	1,06E00	2,51E-02	1,342	C3,CALML5,MMP2,PRKAR2A,RAP1B
Cardiac Hypertrophy Signaling	7,09E-01	1,92E-02	1,342	CALML5,GNB1,HSPB1,PRKAR2A,RAP1B
Dendritic Cell Maturation	0	8,39E-03	1,342	COL18A1,COL1A1,COL1A2,COL3A1,HLA-B
Epithelial Adherens Junction Signaling	4,75E00	6,37E-02	1,265	CDH2,CFL1,FGFR1,MST1,RAP1B,VCL,YWHAE,YWHAG,YWHAQ,YWHAZ
Phagosome Formation	5,16E-01	1,44E-02	1,265	C3,CFL1,FN1,ITGA2,ITGA5,ITGB1,MYH9,RAP1B,TLN1,VTN
Leukocyte Extravasation Signaling	6,28E00	6,74E-02	1,155	ACTB,ACTN1,CD44,ITGA2,ITGB1,MMP1,MMP2,MMP3,MSN,RAP1B,TIMP1,TIMP2,VCL
PI3K/AKT Signaling	5,32E00	0,06	1,155	HSP90AA1,HSP90AB1,HSP90B1,ILK,ITGA2,ITGA5,ITGB1,RAP1B,YWHAE,YWHAG,YWHAQ,YWHAZ
Signaling by Rho Family GTPases	2,34E00	3,37E-02	1,134	ACTB,CDH2,CFL1,GNB1,ITGA2,ITGA5,ITGB1,MSN,VIM
Oxytocin Signaling Pathway	1,3E00	2,48E-02	1,134	CALML5,EEF2,GNB1,HSPB1,MYH9,PRKAR2A,RAP1B
Acute Phase Response Signaling	1,42E01	1,14E-01	1,000	A2M,AHSG,ALB,C1R,C3,C4A/C4B,C5,C9,CP,F2,FGB,FN1,ITIH2,ITIH3,ITIH4,PLG,RAP1B,RBP4,SERPIND1,SERPINE1,SERPINF1
Apelin Liver Signaling Pathway	3,57E00	1,48E-01	1,000	COL18A1,COL1A1,COL1A2,COL3A1
Coronavirus Replication Pathway	2,72E00	8,89E-02	1,000	TUBA4A,TUBB,TUBB1,TUBB4B
Xenobiotic Metabolism AHR Signaling Pathway	1,71E00	4,6E-02	1,000	ALDH9A1,HSP90AA1,HSP90AB1,HSP90B1
Protein Kinase A Signaling	1,61E00	2,43E-02	1,000	CALML5,FLNA,GNB1,PRKAR2A,PYGL,RAP1B,YWHAE,YWHAG,YWHAQ,YWHAZ
Role of NFAT in Cardiac Hypertrophy	5,66E-01	1,79E-02	1,000	CALML5,GNB1,PRKAR2A,RAP1B
Opioid Signaling Pathway	3,77E-01	1,43E-02	1,000	CALML5,GNB1,PRKAR2A,RAP1B
Senescence Pathway	3,28E-01	1,34E-02	1,000	CALML5,CAT,RAP1B,SERPINE1
CTLA4 Signaling in Cytotoxic T Lymphocytes	0	6,58E-03	1,000	HLA-B,ITGA2,ITGB1,RAP1B
CDC42 Signaling	0	8,68E-03	1,000	CFL1,HLA-B,ITGA2,ITGA5,ITGB1
G-Protein Coupled Receptor Signaling	0	5,69E-03	1,000	CALML5,GNB1,PRKAR2A,RAP1B
RAC Signaling	2,24E00	4,38E-02	0,816	CD44,CFL1,ITGA2,ITGA5,ITGB1,RAP1B
Xenobiotic Metabolism PXR Signaling Pathway	1,58E00	3,12E-02	0,816	ALDH9A1,CAT,HSP90AA1,HSP90AB1,HSP90B1,PRKAR2A
Colorectal Cancer Metastasis Signaling	1,38E00	2,58E-02	0,816	GNB1,LRP1,MMP1,MMP2,MMP3,PRKAR2A,RAP1B
Multiple Sclerosis Signaling Pathway	1,32E00	2,7E-02	0,816	C3,C5,C7,C8B,C9,HLA-B
Estrogen Receptor Signaling	2,02E00	2,69E-02	0,707	CFL1,GNB1,HSP90AA1,HSP90AB1,HSP90B1,IGF2R,MMP1,MMP2,MMP3,PRKAR2A,RAP1B
ILK Signaling	5,29E00	5,97E-02	0,632	ACTB,ACTN1,CFL1,DSP,FLNA,FN1,ILK,ITGB1,MYH9,NACA,VCL,VIM
MSP-RON Signaling In Cancer Cells Pathway	5,18E00	7,14E-02	0,632	FLNA,HGFAC,ITGB1,MST1,RAP1B,VIM,YWHAE,YWHAG,YWHAQ,YWHAZ
Tumor Microenvironment Pathway	6,65E00	7,26E-02	0,577	ARG1,CD44,COL1A1,COL1A2,COL3A1,FN1,HLA-B,ITGA5,MMP1,MMP2,MMP3,RAP1B,TNC
HIF1α Signaling	5,91E00	6,25E-02	0,577	HSP90AA1,HSPA1A/HSPA1B,HSPA5,HSPA8,LDHB,MMP1,MMP2,MMP3,PKM,RAN,RAP1B,SERPINE1,VIM
Neutrophil Extracellular Trap Signaling Pathway	4,46E00	3,87E-02	0,500	C5,CASP14,COL12A1,COL15A1,COL18A1,COL1A1,COL1A2,COL3A1,COL5A1,COL6A1,COL6A2,COL6A3,ITGA2,ITGB1,LTF,MMP2
BAG2 Signaling Pathway	1,32E01	1,79E-01	0,447	ANXA2,HSP90AA1,HSPA1A/HSPA1B,HSPA5,HSPA8,PSMA1,PSMA3,PSMA4,PSMA5,PSMA6,PSMA7,PSMB2,PSMB3,PSMB5,PSMD2
Complement System	8,02E00	2,16E-01	0,447	C1R,C3,C4A/C4B,C5,C7,C8B,C9,CFI
ERK5 Signaling	2,74E00	6,76E-02	0,447	RAP1B,YWHAE,YWHAG,YWHAQ,YWHAZ
NRF2-mediated Oxidative Stress Response	2,68E00	3,8E-02	0,447	ACTB,CAT,FTH1,HSP90AA1,HSP90AB1,HSP90B1,PPIB,RAP1B,UBB
Inhibition of Matrix Metalloproteases	9,22E00	2,31E-01	0,378	A2M,HSPG2,LRP1,MMP1,MMP2,MMP3,THBS2,TIMP1,TIMP2
HOTAIR Regulatory Pathway	3,14E00	4,91E-02	0,378	CD44,COL1A1,COL1A2,COL3A1,MMP1,MMP2,MMP3,VIM
Ferroptosis Signaling Pathway	3,01E00	5,3E-02	0,378	ARF3,FTH1,H2BC12,HSPB1,PEBP1,RAP1B,TFRC
Role Of Chondrocytes In Rheumatoid Arthritis Signaling Pathway	2,84E00	4,96E-02	0,378	FN1,ITGA5,ITGB1,LRP1,MMP1,MMP2,MMP3
MicroRNA Biogenesis Signaling Pathway	2,16E00	3,74E-02	0,378	HSP90AA1,HSP90AB1,HSP90B1,HSPA8,RAN,RAP1B,ST13
Coagulation System	9,68E00	2,57E-01	0,333	A2M,F10,F2,F5,FGB,PLG,SERPINC1,SERPIND1,SERPINE1
Natural Killer Cell Signaling	4,61E00	5,56E-02	0,302	CFL1,COL18A1,COL1A1,COL1A2,COL3A1,HLA-B,HSPA1A/HSPA1B,HSPA5,HSPA8,ITGB1,RAP1B
IL-12 Signaling and Production in Macrophages	5,3E00	5,51E-02	0,277	ALB,APOB,APOE,C3,CALML5,CLU,COL18A1,COL1A1,COL1A2,COL3A1,MST1,RBP4,THBS1
Role Of Osteoclasts In Rheumatoid Arthritis Signaling Pathway	6,76E00	5,5E-02	0,243	CALML5,COL12A1,COL15A1,COL18A1,COL1A1,COL1A2,COL3A1,COL5A1,COL6A1,COL6A2,COL6A3,GSN,ITGA5,MMP1,MMP2,MMP3,RAP1B
Pulmonary Fibrosis Idiopathic Signaling Pathway	1,37E01	7,98E-02	0,000	ACTB,CDH2,COL12A1,COL15A1,COL18A1,COL1A1,COL1A2,COL3A1,COL5A1,COL6A1,COL6A2,COL6A3,F2,FGFR1,FN1,ILK,ITGA2,ITGB1,MMP1,MMP2,MMP3,PLG,RAP1B,SERPINE1,THBS1,VIM
14-3-3-mediated Signaling	5,56E00	7,87E-02	0,000	RAP1B,TUBA4A,TUBB,TUBB1,TUBB4B,VIM,YWHAE,YWHAG,YWHAQ,YWHAZ
Neuroprotective Role of THOP1 in Alzheimer's Disease	4,02E00	6,61E-02	0,000	APP,C1R,HGFAC,HLA-B,HTRA1,PLG,PRKAR2A,YWHAE
HIPPO signaling	3,27E00	6,98E-02	0,000	CD44,MST1,YWHAE,YWHAG,YWHAQ,YWHAZ
Role of PKR in Interferon Induction and Antiviral Response	2,26E00	4,41E-02	0,000	HSP90AA1,HSP90AB1,HSP90B1,HSPA1A/HSPA1B,HSPA5,HSPA8
luction of Nitric Oxide and Reactive Oxygen Species in Macropha	2,12E00	3,66E-02	0,000	ALB,APOB,APOE,CAT,CLU,RAP1B,RBP4
IL-17 Signaling	1,62E00	3,21E-02	0,000	HSP90AA1,HSP90AB1,HSP90B1,MMP2,MMP3,RAP1B
NOD1/2 Signaling Pathway	1,61E00	3,17E-02	0,000	HSP90AA1,HSP90AB1,HSP90B1,HSPA1A/HSPA1B,HSPA5,HSPA8
on Of The Epithelial Mesenchymal Transition By Growth Factors	1,58E00	3,12E-02	0,000	CDH2,FGFR1,MMP1,MMP2,RAP1B,VIM
PPAR Signaling	1,42E00	3,74E-02	0,000	HSP90AA1,HSP90AB1,HSP90B1,RAP1B
Telomerase Signaling	1,41E00	3,7E-02	0,000	HSP90AA1,HSP90AB1,HSP90B1,RAP1B
IL-4 Signaling	1,38E00	2,08E-02	0,000	ARG1,COL12A1,COL15A1,COL18A1,COL1A1,COL1A2,COL3A1,COL5A1,COL6A1,COL6A2,COL6A3,RAP1B
RHOA Signaling	1,23E00	3,23E-02	0,000	ACTB,CFL1,MSN,PFN1
GNRH Signaling	1,12E00	2,62E-02	0,000	CALML5,GNB1,MMP2,PRKAR2A,RAP1B
SNARE Signaling Pathway	1,11E00	2,94E-02	0,000	CALML5,HSPA8,MYH9,PRKAR2A
Xenobiotic Metabolism CAR Signaling Pathway	7,24E-01	2,09E-02	0,000	ALDH9A1,HSP90AA1,HSP90AB1,HSP90B1
HER-2 Signaling in Breast Cancer	5,54E-01	1,76E-02	0,000	ARF3,ITGB1,MMP2,RAP1B
Breast Cancer Regulation by Stathmin1	3,97E-01	1,35E-02	0,000	GNB1,MMP2,PRKAR2A,RAP1B,TUBA4A,TUBB,TUBB1,TUBB4B
S100 Family Signaling Pathway	1,39E00	1,94E-02	-0,258	AHNAK,ANXA1,ANXA2,APP,CALML5,FGFR1,ILK,MMP1,MMP2,MMP3,MYH9,NCL,PLG,PRKAR2A,SERPINF1
LXR/RXR Activation	8,61E00	1,06E-01	-0,277	AHSG,ALB,APOB,APOE,C3,C4A/C4B,C9,CLU,GC,ITIH4,RBP4,SERPINF1,VTN
Immunogenic Cell Death Signaling Pathway	5,93E00	0,1	-0,333	ANXA1,CALR,HSP90AA1,HSP90AB1,HSP90B1,HSPA1A/HSPA1B,HSPA5,HSPA8,LRP1
Glioma Invasiveness Signaling	4,61E00	9,59E-02	-0,378	CD44,MMP2,PLG,RAP1B,TIMP1,TIMP2,VTN
Unfolded protein response	4,02E00	7,78E-02	-0,378	CALR,HSP90B1,HSPA1A/HSPA1B,HSPA5,HSPA8,P4HB,PDIA6
Pulmonary Healing Signaling Pathway	2,02E00	3,52E-02	-0,378	CFL1,FGFR1,MMP1,MMP2,MMP3,RAP1B,THBS1
Chronic Myeloid Leukemia Signaling	1,33E00	2,52E-02	-0,378	CALML5,HSP90AA1,HSP90AB1,HSP90B1,ITGB1,RAN,RAP1B
Inhibition of ARE-Mediated mRNA Degradation Pathway	9,03E00	9,26E-02	-0,447	PRKAR2A,PSMA1,PSMA3,PSMA4,PSMA5,PSMA6,PSMA7,PSMB2,PSMB3,PSMB5,PSMD2,YWHAE,YWHAG,YWHAQ,YWHAZ
eNOS Signaling	3,27E00	5,13E-02	-0,447	CALML5,HSP90AA1,HSP90AB1,HSP90B1,HSPA1A/HSPA1B,HSPA5,HSPA8,PRKAR2A
Dilated Cardiomyopathy Signaling Pathway	2,05E00	0,04	-0,447	ACTB,ILK,LAMA4,LMNA,MYH9,PRKAR2A
Nitric Oxide Signaling in the Cardiovascular System	1,86E00	4,17E-02	-0,447	CALML5,HSP90AA1,HSP90AB1,HSP90B1,PRKAR2A
PPARα/RXRα Activation	1,55E00	3,08E-02	-0,447	CAND1,HSP90AA1,HSP90AB1,HSP90B1,PRKAR2A,RAP1B
Human Embryonic Stem Cell Pluripotency	1,05E00	2,49E-02	-0,447	FGFR1,HSP90AA1,HSP90AB1,HSP90B1,RAP1B
CREB Signaling in Neurons	0	9,88E-03	-0,447	CALML5,FGFR1,GNB1,IGF2R,PRKAR2A,RAP1B
Pathogen Induced Cytokine Storm Signaling Pathway	3,85E00	3,77E-02	-0,535	C3,C5,COL12A1,COL15A1,COL18A1,COL1A1,COL1A2,COL3A1,COL5A1,COL6A1,COL6A2,COL6A3,FGB,FTH1

GP6 Signaling Pathway	1,51E01	1,5E-01	-0,688	CALML5,COL12A1,COL15A1,COL18A1,COL1A1,COL1A2,COL3A1,COL5A1,COL6A1,COL6A2,COL6A3,FGB,LAMA4,LAMA5,LAMB1,LAMB2,LAMC1,RAP1B,TLN1
Role of JAK family kinases in IL-6-type Cytokine Signaling	3,47E00	7,59E-02	-0,816	HSP90AA1,HSP90AB1,HSP90B1,MMP1,SERPINA7,TIMP1
Semaphorin Neuronal Repulsive Signaling Pathway	2,05E00	0,04	-0,816	CD44,CFL1,ITGA2,ITGA5,ITGB1,PRKAR2A
ID1 Signaling Pathway	1,49E00	2,99E-02	-0,816	APP,FGFR1,FN1,MMP2,RAP1B,VIM
Role Of Osteoblasts In Rheumatoid Arthritis Signaling Pathway	1,16E00	2,46E-02	-0,816	COL1A1,IGF2R,LRP1,MMP1,MMP2,MMP3
Myelination Signaling Pathway	3,83E00	3,98E-02	-0,832	CALML5,FGFR1,IGF2R,ILK,ITGA2,ITGB1,LAMA4,LAMA5,LAMB1,LAMB2,LAMC1,PRKAR2A,RAP1B
PTEN Signaling	2,67E00	4,64E-02	-1,134	FGFR1,IGF2R,ILK,ITGA2,ITGA5,ITGB1,RAP1B
CLEAR Signaling Pathway	1,28E00	2,46E-02	-1,134	FGFR1,IGF2R,RAP1B,YWHAE,YWHAG,YWHAQ,YWHAZ
CDK5 Signaling	3,36E00	6,09E-02	-1,342	ITGA2,ITGB1,LAMA5,LAMB1,LAMC1,PRKAR2A,RAP1B
Systemic Lupus Erythematosus In T Cell Signaling Pathway	0	7,78E-03	-1,342	CASP14,CD44,HLA-B,MSN,RAP1B
RHOGDI Signaling	3,53E00	4,55E-02	-1,414	ACTB,CD44,CDH2,CFL1,GNB1,ITGA2,ITGA5,ITGB1,MSN,MYH9
Chaperone Mediated Autophagy Signaling Pathway	5,06E00	3,42E-02	-1,706	APP,EEF1A1,HSP90AA1,HSP90AB1,HSP90B1,HSPA1A/HSPA1B,HSPA5,HSPA8,IDH1,MMP1,MMP2,MMP3,MST1,PSMA1,PSMA3,PSMA4,PSMA5,PSMA6,PSMA7,PSMB2,PSMB3,PSMB5

Supplemental table 2

Symbol	Entrez Gene Name	Expr Fold Change	Expr p-value	Expected
ALDOA	aldolase, fructose-bisphosphate A	1,109	2,58E-01	Up
ENO1	enolase 1	1,043	5,72E-01	Up
GAPDH	glyceraldehyde-3-phosphate dehydrogenase	1,014	5,90E-01	Up
PFKL	phosphofructokinase, liver type	1,092	3,97E-01	Up
PGAM2	phosphoglycerate mutase 2	1,260	9,12E-02	Up
PGK1	phosphoglycerate kinase 1	1,297	4,43E-01	Up
PKM	pyruvate kinase M12	1,211	2,41E-01	Up
TPI1	triosephosphate isomerase 1	1,250	1,32E-01	Up
